# CABOPRE: Evaluating Perioperative Cabozantinib’s Efficacy and Biomarkers for Cytoreductive Nephrectomy in Advanced Renal Cell Carcinoma

**DOI:** 10.3390/cancers18193216

**Published:** 2026-10-05

**Authors:** Guillermo de Velasco, Teresa Alonso-Gordoa, Juan Francisco Rodríguez-Moreno, Ignacio Duran, Lucía Carril Ajuria, Pablo Álvarez Ballesteros, Enrique González-Billalabeitia, José I. Domínguez, Ana García-Casas, Enrique Pérez-Navarro, Sara Sánchez-Redondo, Javier Molina-Cerrillo, Felix Guerrero-Ramos, Ray Manneh Kopp, Cecilia Marinas, Jesús M. Paramio, Héctor Peinado, Daniel Castellano, Marta Dueñas

**Affiliations:** 1 Medical Oncology/Urology Department, Hospital Universitario 12 de Octubre, 28041 Madrid, Spain; pabloteros@gmail.com (P.Á.B.); egbillalabeitia@salud.madrid.org (E.G.-B.); felix.guerrero@salud.madrid.org (F.G.-R.); daniel.castellano@salud.madrid.org (D.C.); 2 Instituto de Investigación Sanitaria Hospital 12 de Octubre (Imas12), 28041 Madrid, Spain; jignaciodominguez.imas12@h12o.es (J.I.D.); anagarciacasas.imas12@h12o.es (A.G.-C.); cemarinas.imas12@h12o.es (C.M.); jesusm.paramio@ciemat.es (J.M.P.); marta.duenas@ciemat.es (M.D.); 3 Medical Oncology Department, Hospital Universitario Ramón y Cajal, 28034 Madrid, Spain; talonsogordoa@gmail.com (T.A.-G.); javier.molinace@gmail.com (J.M.-C.); 4 IRYCIS ( Instituto Ramón y Cajal de Investigación Sanitaria), 28034 Madrid, Spain; 5 Instituto de Investigación Sanitaria HM Hospitales, 28015 Madrid, Spain; jfrodriguez@hmhospitales.com; 6 Facultad de Medicina, Universidad San Pablo CEU, 28925 Madrid, Spain; 7 Oncology Department, Hospital Universitario Marques de Valdecilla, IDIVAL, 39008 Cantabria, Spain; ignaciojose.duran@scsalud.es; 8 Medical Oncology Department, CHU Saint Pierre, 1000 Brussels, Belgium; lucia.carril@stpierre-bru.be; 9 Departamento de Ingeniería de la Información y las Comunicaciones, Universidad de Murcia, 30100 Murcia, Spain; enrique.perez2@um.es; 10 Microenvironment and Metastasis Laboratory, Molecular Oncology Programme, Spanish National Cancer Research Center (CNIO), 28029 Madrid, Spain; ssanchezr@cnio.es (S.S.-R.); hpeinado@cnio.es (H.P.); 11 Medical Oncology, Sociedad de Oncología y Hematología del Cesar, Valledupar 200001, Colombia; ray.manneh@sohec.com.co; 12 Molecular Oncology Unit, CIEMAT ( Centro de Investigaciones Energéticas, Medioambientales y Tecnológicas), 28040 Madrid, Spain

**Keywords:** metastatic renal cell carcinoma, cabozantinib, cytoreductive nephrectomy, biomarkers

## Abstract

Background/Objectives: Despite advances in metastatic renal cell carcinoma (mRCC), long-term survival is poor and biomarkers are lacking. Optimal integration of cytoreductive nephrectomy (CN) in the current era remains controversial. CABOPRE, the first phase II trial assessing perioperative cabozantinib in mRCC patients eligible for CN, aimed to determine its effects in this setting, integrating tumor outcomes with molecular profiling. Methods: CABOPRE (NCT06377722) is a multicentre, single-arm, phase II study conducted in Spanish academic hospitals. Patients with resectable metastatic clear cell RCC (ccRCC) eligible for post-treatment CN received perioperative cabozantinib (60 mg/day for 12 weeks; *n* = 18). The primary endpoint was the objective response rate (ORR) at 12 weeks assessed by RECIST v1.1. Exploratory analyses included longitudinal plasma miRNA and spatial transcriptomic profiling to identify candidate biomarkers. The trial closed early because of slow accrual, after enrolling 18 of 50 planned patients and without completing the 26-patient first stage of the Simon two-stage design; the planned interim analysis was therefore never performed. Results: Because the two-stage design was not completed, the primary endpoint could not be formally evaluated against the prespecified decision rule. Among 15 evaluable patients, the objective response rate was 26.7% (95% CI, 7.8–55.1), with stable disease in 10 (66.7%) and progressive disease in 1 (6.7%). Cabozantinib demonstrated a manageable safety profile (Grade 1–2 adverse events). In exploratory biomarker analyses, *miR-126-3p* upregulation and *VEGFA/CCND1* downregulation were associated with tumor shrinkage, and the adhesion molecules *ITGAV* and *ICAM1* were upregulated in these patients. Because no direct vascular measurements were performed, these changes are described as treatment-associated and hypothesis-generating rather than as predictors of response or evidence of vascular normalization. Conclusions: The observed response rate was close to the 25% null rate, and the prespecified efficacy rule could not be applied. CABOPRE provides preliminary evidence that perioperative cabozantinib is active and represents a viable backbone for neoadjuvant combination strategies in selected patients with potentially resectable mRCC. The identified molecular heterogeneity and stromal-immune signatures provide a biological rationale for the prospective evaluation of cabozantinib-IO combinations in this setting.

## Simple Summary

Patients with advanced kidney cancer that has spread to other organs often face a poor long-term survival outlook, and doctors lack clear tools to predict who will benefit most from specific treatments. Additionally, the role of surgically removing the primary kidney tumor after initial drug therapy remains highly debated. This clinical trial aimed to evaluate the safety and effectiveness of using the targeted drug cabozantinib before surgery to shrink bulky tumors and make them easier to remove. The study successfully showed that this approach shrinks tumors in most patients with manageable side effects. Furthermore, by analyzing blood and tumor samples, researchers identified specific molecular signals linked to treatment success. These findings help the scientific community better understand how this specific therapy reshapes tumors, guiding future treatment designs.

## 1. Introduction

The management of metastatic renal cell carcinoma (mRCC) has been reshaped by anti-angiogenic tyrosine kinase inhibitors (TKI) and immune checkpoint inhibitors (ICIs). Despite advances in systemic therapy, long-term survival remains poor, with fewer than 40% of patients surviving beyond five years even with contemporary ICI-based regimens [1,2,3,4]. A critical unresolved challenge is the absence of robust biomarkers to guide treatment, underscoring an urgent need for precision-driven strategies.

Cytoreductive nephrectomy (CN) has historically been considered a cornerstone of mRCC management, particularly in the cytokine era. However, landmark trials such as CARMENA and SURTIME demonstrated the non-inferiority of upfront systemic therapy (e.g., sunitinib) and hinted at potential benefits of deferred CN in patients who achieve a response [5,6].

This paradigm shift raises a pivotal question: Can CN retain therapeutic relevance within this complex era of systemic treatments, and if so, for which patients? Meta-analyses suggest deferred CN may optimize outcomes, particularly for patients without progression on systemic therapy [7]. These results support the notion that patients requiring systemic treatment might avoid surgery initially [6,8,9]. Nonetheless, CN could still be a viable option for selected patients who respond to systemic therapy and for good-risk patients who do not require additional treatment [10,11,12]. Optimal management of the primary tumor in the current era of immune combinations remains undefined [12,13,14].

Prior to the widespread adoption of ICIs and combination therapies, cabozantinib monotherapy emerged as a significant advancement in the treatment of mRCC, particularly for patients who had progressed on prior therapies. As a potent multi-target TKI inhibiting VEGFR, MET, and AXL, it demonstrates significantly higher response rates compared to sunitinib in patients with intermediate and poor IMDC risk, with the potential to enhance resectability through mechanisms of vascular normalization and stromal remodeling [15,16,17]. Notably, cabozantinib remains a guideline-recommended therapy (NCCN) for intermediate- and poor-risk mRCC, particularly when ICIs are contraindicated, highlighting its continued relevance in contemporary treatment paradigms [18].

Importantly, evaluating cabozantinib as a monotherapy in the perioperative window provides a clean, unconfounded biological model to dissect the specific mechanisms of TKI-induced tumor cytoreduction and tissue remodeling, free from the overlapping signatures of concurrent immunotherapy.

Here we present the clinical and translational results of CABOPRE (EudraCT 2018-001201-93), the first phase II trial evaluating perioperative cabozantinib (60 mg daily) in mRCC patients eligible for CN. By integrating tumor response data with longitudinal plasma miRNA profiling and spatial transcriptomics, this study offers a mechanistic characterization of cabozantinib in the perioperative setting that is unlikely to be reproduced in the current era of ICI-based combinations, and may inform the rational design of future neoadjuvant strategies.

## 2. Materials and Methods

### 2.1. Study Design and Participants

**Figure 1:**
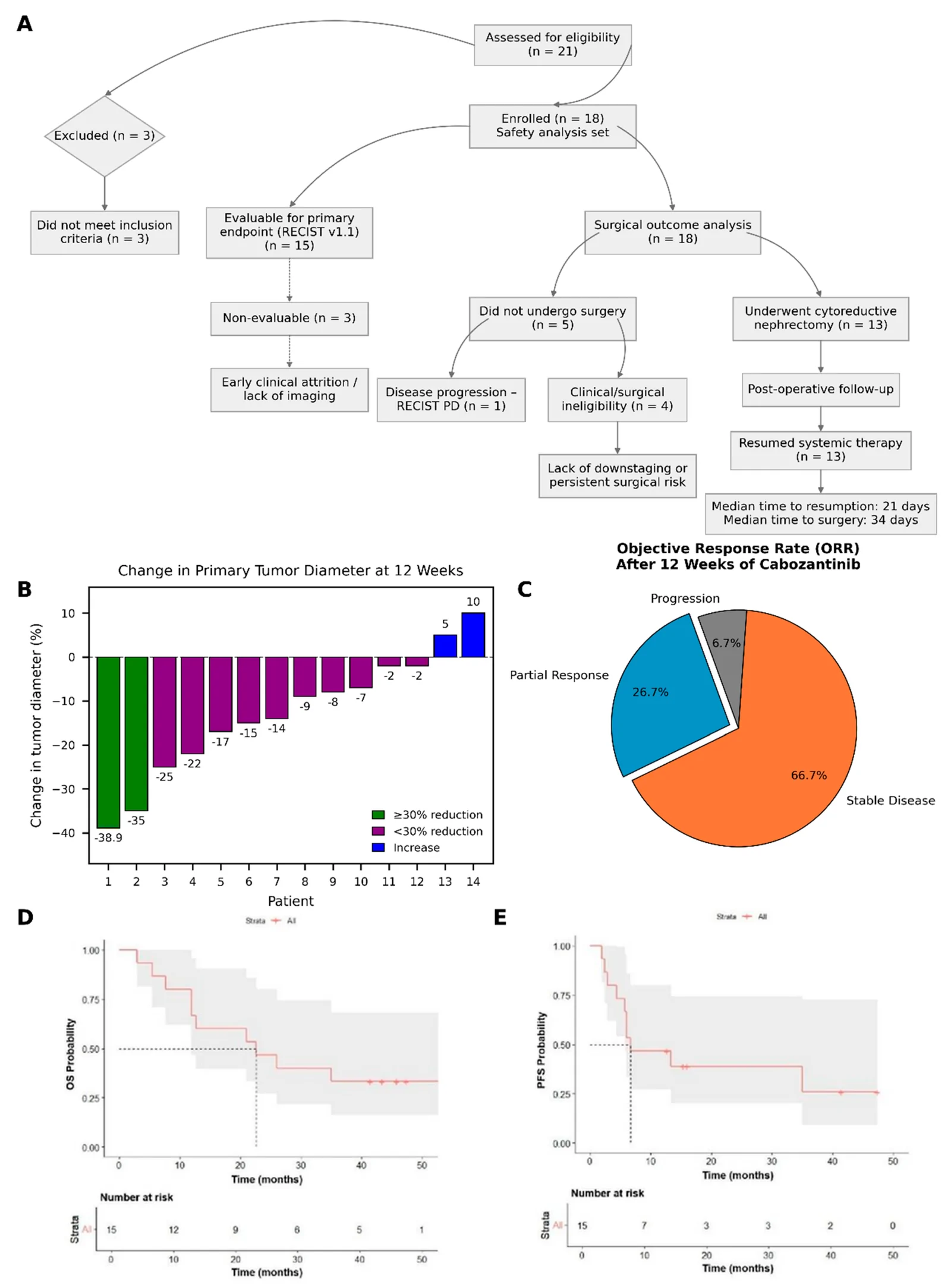
Clinical efficacy outcomes. (**A**) Study design of the CABOPRE Trial. (**B**) Waterfall plot of the percentage change in primary tumor diameter at 12 weeks. This panel assesses the local antitumor activity of cabozantinib on the primary tumor, which is the determinant of surgical resectability, it is not a RECIST assessment (*n* = 15). (**C**) Objective Response Rate (ORR) after 12 Weeks of Cabozantinib by RECIST criteria v1.1. ORR based on the sum of diameters of all target lesions. Of the 15 patients evaluated, four (26.7%) underwent a partial response, by RECIST criteria. (**D**,**E**) Kaplan–Meier curves depicting overall survival (OS) and progression-free survival (PFS), respectively, in patients treated with cabozantinib. The Y-axis represents the probability of survival, ranging from 0 to 1. The X-axis represents time in months. The lower panel shows the number of patients at risk at each time point.

The CABOPRE trial (EudraCT: 2018-001201-93; NCT06377722) is a multicenter, single-arm, phase II study evaluating perioperative cabozantinib (60 mg/day for 12 weeks) in patients with metastatic clear-cell renal cell carcinoma (ccRCC) deemed resectable by multidisciplinary consensus (Figure 1A). Eligible patients had histologically confirmed ccRCC, ECOG performance status 0–1, and adequate hematologic/organ function. Exclusion criteria included prior systemic therapy, active malignancies (<3 years), or tumors involving the gastrointestinal tract or tracheobronchial tree.

Patients received cabozantinib (60 mg/day) for three 28-day cycles. CN was performed 28–42 days posttreatment, with cabozantinib resumed ≥2 weeks postoperatively pending wound healing and investigator approval, and continued daily thereafter (the complete protocol is available in the appended file, File S1).

The primary endpoint was objective response rate (ORR) at 12 weeks (RECIST v1.1). Key secondary endpoints included successful CN rate, progression-free survival (PFS), overall survival (OS), safety/tolerability, and biomarker changes pre- to post-treatment. Clinical responders were strictly defined by RECIST v1.1 criteria (PR/CR).

For exploratory biomarker and Digital Spatial Profiling (DSP) analyses, a sensitive threshold of 10% tumor shrinkage was utilized to capture molecular changes in patients deriving clinical benefit below the formal PR threshold. This cut-off was defined post hoc and was not prespecified in the protocol. It was adopted because only four patients achieved a RECIST partial response, too few to compare molecular profiles between RECIST-defined categories, and because the short neoadjuvant window (12 weeks) may allow biological activity to precede a formal RECIST response. All analyses based on this definition are exploratory and hypothesis-generating.

Tumor shrinkage (any reduction from baseline) was explored descriptively.

### 2.2. Statistical Analysis

Simon’s two-stage design (α = 0.05, 90% power) enrolled 26 patients in stage 1; expansion to stage 2 (total *n* = 50) required ≥8 responses. Continuous variables are reported as median (IQR), categorical variables as frequencies (%), and survival outcomes via Kaplan–Meier estimates (95% CI). Paired *t*-tests or Wilcoxon signed-rank tests analyzed miRNA/DSP changes. For clinical efficacy analysis, responders (complete response [CR]/partial response [PR]) versus non-responders (stable disease [SD]/progressive disease [PD]) were compared using logistic regression (SAS v9.4; SAS Institute Inc., Cary, NC, USA).

Sample size was determined using Simon’s two-stage optimal design. The null hypothesis was an objective response rate of 25%, based on the activity of cabozantinib reported in the METEOR trial, tested against an alternative of 45%, with a one-sided type I error of 0.05 and 90% power. Under these assumptions, 26 patients were required in the first stage and 50 in total. The trial would be stopped for futility if 7 or fewer responses were observed among the first 26 patients; if more than 7 responses were observed, accrual would continue to 50, and 18 or more responses in total would be required to consider the regimen worthy of further evaluation. The expected sample size under the null hypothesis was 33.6 patients, with a 0.69 probability of early termination.

Recruitment closed after 18 patients because of slow accrual and the rapid evolution of the first-line standard. The first stage was therefore not completed, the planned interim analysis was not performed, and the prespecified decision rule was never applied. All efficacy analyses are consequently descriptive. Response proportions are reported with exact 95% confidence intervals. Progression-free and overall survival were estimated by the Kaplan–Meier method. Subgroup comparisons by IMDC risk category and by receipt of cytoreductive nephrectomy were unplanned and are reported for completeness in Supplementary Table S3; they were not adjusted for multiplicity and should be interpreted as exploratory. Two analysis populations were defined. The full analysis set comprised all 18 patients who received at least one dose of cabozantinib and was used for safety, survival and the intention-to-treat response rate, in which the three patients without a 12-week assessment were counted as non-responders. The per-protocol (evaluable) population comprised the 15 patients with a 12-week tumor assessment and was used for the primary response analysis. No imputation was performed.

### 2.3. Biomarker Analysis and Spatial Transcriptomic

Longitudinal biomarker studies were performed on paired patient samples, when sample quality and availability thresholds were met. To investigate molecular mechanisms of response, two complementary approaches were employed.

First, plasma and tumor-derived exosomal RNA were collected pre/post cabozantinib for microRNA (miRNA) profiling. Circulating miRNAs were isolated from plasma and tumor-derived exosomes using ultracentrifugation. Genome-wide miRNA expression was quantified via the NanoString nCounter^®^ platform (NanoString Technologies, Inc., Seattle, WA, USA), focusing on angiogenesis- and immune-related miRNAs. Second, formalin-fixed, paraffin-embedded (FFPE) tumor samples underwent spatially resolved transcriptomic analysis using the NanoString GeoMx^®^ Digital Spatial Profiler to assess spatially resolved gene expression through the IO Proteome (immune microenvironment) and the PanCancer Pathways panels (oncogenic signaling).

For the DSP protocol, 4 μm FFPE tumor sections were deparaffinized, rehydrated, and subjected to antigen retrieval in pH 9.0 buffer (95 °C, 20 min). Slides were co-stained with fluorescent markers for tissue morphology—pan-cytokeratin (panCK, tumor epithelium), CD45 (immune cells), and Syto13 (nuclear counterstain)—alongside photocleavable RNA-targeted probes from the GeoMx^®^ RNA Immune Pathways Panel (83 genes). High-resolution digital maps were generated, and a uropathologist identified regions of interest (ROIs) based on H&E-stained reference slides. For tissue microarrays (TMAs), two ROIs (660 μm diameter) were selected per core, a size specifically optimized to balance spatial resolution with a representative assessment of tumor heterogeneity. In whole-tissue sections, ROIs focused on immune-enriched areas (panCK+/CD45+ colocalization) to prioritize the evaluation of cabozantinib’s impact on immune-stromal crosstalk.

Within each ROI, molecular compartments were defined using fluorescence colocalization. The tumor compartment was identified by panCK+ epithelial cells, while the stromal compartment comprised panCK−/Syto13+ nuclei in non-epithelial regions. Immune infiltrates were specifically characterized as CD45+ cells located within either the tumor or stromal areas. UV light was applied to release oligonucleotide tags from each compartment, which were collected and hybridized to optical barcodes for digital quantification using the NanoString nCounter^®^ system. Data normalization followed a two-step process: (a) Technical normalization: Adjusted for batch effects using External RNA Controls Consortium (ERCC) spike-in synthetic RNAs. (b) Biological normalization: Scaled to the geometric mean of four housekeeping genes (POLR2A, RAB7A, SDHA, OAZ1), which were selected for stable expression in ccRCC and validated in prior ccRCC studies to minimize normalization bias.

For exploratory biomarker and DSP analyses, patients were classified post hoc as molecular responders (≥10% reduction in tumor size at 12 weeks) or non-responders (<10%). This threshold was not prespecified; it was adopted because only four patients achieved a RECIST partial response, which would have left groups too small for comparison. The rationale and limitations of this cut-off are discussed below. Finally, differential expression analysis compared molecular responders (≥10% tumor shrinkage) versus non-responders (<10% shrinkage) using NanoString nSolver^®^ software (v4.0, NanoString Technologies, Inc., Seattle, WA, USA) was used. Spatial gene expression patterns (e.g., *VEGFA, CCND1, ITGAV*) were correlated with clinical outcomes (PFS, OS) and miRNA profiles. The number of patients with both paired plasma and paired tissue samples was insufficient for integration at the patient level. Unless otherwise stated, tests were two-sided with a nominal significance level of 0.05. For the miRNA analyses, multiple testing was controlled using the Benjamini–Hochberg false discovery rate and significance was defined as FDR < 0.05. Because of the small number of paired tissue samples, DSP comparisons were not corrected for multiple testing; they are reported with unadjusted *p* values together with a |log2 fold change| > 1 threshold and are exploratory.

## 3. Results

### 3.1. Patient and Treatment Characteristics

Between December 2018 and December 2020, 21 patients with metastatic clear-cell renal cell carcinoma (ccRCC) were screened across three Spanish academic centers. Eighteen patients (median age: 57 years, range: 40–80; male: 67%) met eligibility criteria, including ECOG performance status ≤ 1, resectable disease (validated by multidisciplinary tumor board), and no prior systemic therapy (Figure S1). Sex was defined as a biological attribute (male/female), while gender was not assessed in this study. All baseline characteristics are summarized in Table 1.

The trial was discontinued early due to slow accrual and the rapid adoption of ICI-cabozantinib combinations as the new standard of care. The enrolled cohort represented a high-risk, surgically borderline population: by IMDC criteria, 50% were intermediate-risk and 50% poor-risk, with no favorable-risk patients. Most tumors were locally advanced (T3/T4, 83.3%), and 50% of patients with available nodal data (*n* = 7/14) presented with clinical node-positive disease. Median primary tumor diameter was 96 mm (range: 55–145). All patients had metastatic disease (median: 2 sites; 67% with ≥2 measurable sites), predominantly involving the lungs (72.2%) and bone (38.9%) (Table 1).

### 3.2. Treatment Compliance and Surgical Outcomes

Seventeen patients (94%) completed 12 weeks of neoadjuvant cabozantinib (60 mg daily). One patient discontinued treatment at week 8 due to grade 3 hypertension. CN was performed in 13 patients (72%). Intraoperative complications occurred in one patient (Clavien-Dindo grade IVa) while postoperative grade IV pulmonary embolism was observed in another. Reasons for surgical attrition (*n* = 5) included early disease progression (*n* = 1) and clinical/surgical ineligibility due to lack of sufficient downstaging or persistent surgical risk (*n* = 4) (Figure 1A).

### 3.3. Safety Profile

Treatment-related adverse events (TRAEs) were reported in 94% (17/18) of patients, primarily grade 1–2, including asthenia (66.7%), hypertension (55.6%), and mucositis (38.93%). Grade ≥ 3 TRAEs were infrequent and included asthenia (3 events), decreased appetite (2 events), and transaminases increased, dyspnea, and hypercalcemia (1 event each); no grade 5 events or unexpected safety concerns were observed. Hypertension, the most common high-grade AE by incidence, was grade 1–2 in all cases and was effectively managed with antihypertensive agents. Postoperative recovery was uneventful for most patients, with a median hospital stay of 5 days (range: 3–10 days) (Table S1).

### 3.4. Response and Survival Outcomes

Recruitment was stopped after 18 of 50 planned patients because of slow accrual and the rapid shift in the first-line standard towards immune checkpoint inhibitor combinations. The 26-patient first stage of the Simon design was not completed, the planned interim analysis was not performed, and the prespecified continuation rule was never applied. All efficacy results below should be read as descriptive.

At 12 weeks, 15 of 18 enrolled patients were evaluable for the primary endpoint; three were non-evaluable due to early treatment discontinuation (*n* = 1) or absence of post-baseline imaging (*n* = 2). The ORR was 27% (4/15; all partial responses), with stable disease in 10 patients (67%) and progressive disease in 1 (7%). The median time from cabozantinib discontinuation to surgery was 34 days (range: 27–45), and the median time to systemic therapy resumption was 21 days (range: 14–35) (Figure 1B,C). Counting the three patients without a 12-week assessment as non-responders, the ORR in all 18 treated patients was 22.2% (4/18; 95% CI, 6.4–47.6).

Survival endpoints were analyzed descriptively and should be interpreted as exploratory. At a median follow-up of 16 months (range: 5–36), median progression-free survival (PFS) was 12.7 months (95% CI: 3.4–13.5), and median overall survival (OS) was 24.3 months (95% CI: 5.2–36.3) (Figure 1D,E). Given the sample size, survival by IMDC risk category and by receipt of cytoreductive nephrectomy is not interpreted inferentially in the main text; the corresponding medians, hazard ratios and nominal *p* values are provided in Supplementary Table S3, where the confidence intervals are wide and all comparisons are unplanned and unadjusted for multiplicity.

### 3.5. Biomarker Analysis

#### 3.5.1. miRNA Expression Analysis in Plasma and Tumor-Derived Exosomes

**Figure 2:**
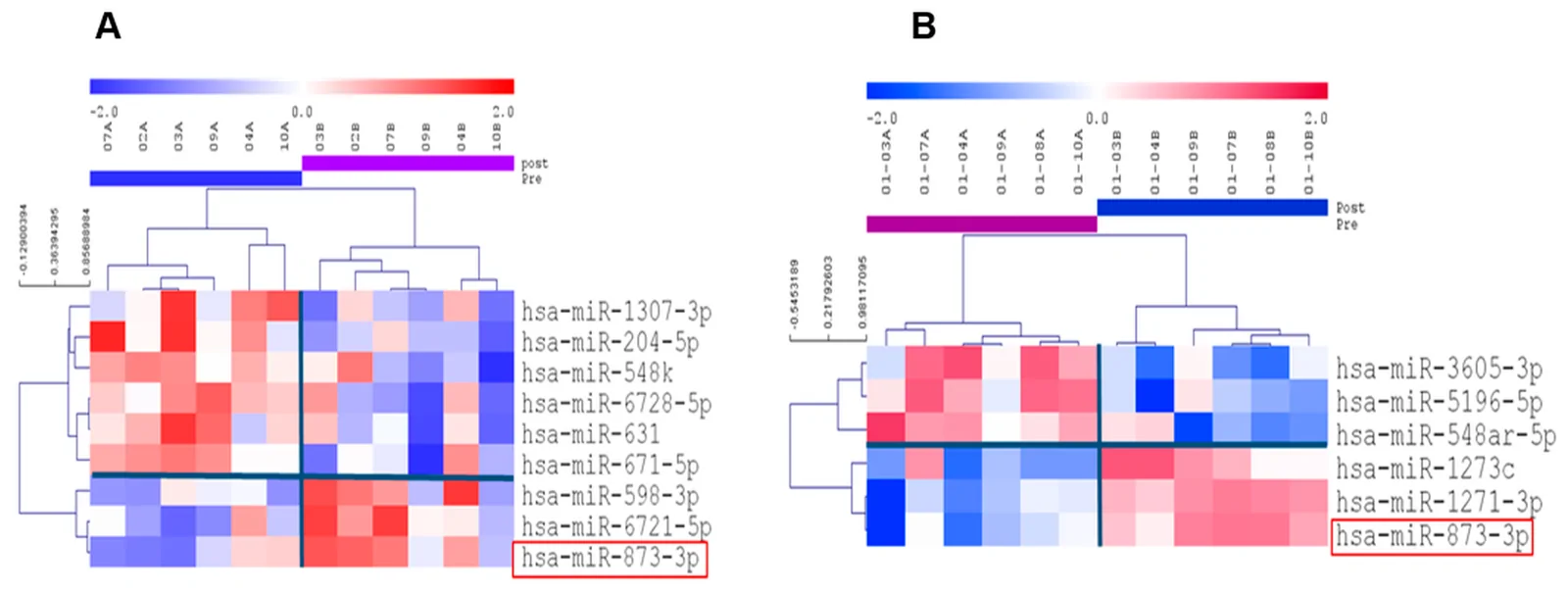
Differential expression of miRNA in plasma and tumour-derived exosomes. (A) Heatmap showing the differential expression of miRNAs before and after cabozantinib treatment in plasma RNA. Each row represents a miRNA and each column represents a sample. Red indicates higher expression, and blue indicates lower expression. A red box highlights *miR-873-3p*, the only miRNA concordantly downregulated in both compartments. (**B**) Heatmap showing the differential expression of miRNAs before and after cabozantinib treatment in tumor-derived exosomal RNA. The layout and color scheme are consistent with Panel (**A**), allowing for a direct comparison of miRNA expression patterns. A red box again highlights *miR-873-3p*.

Paired pre- and post-treatment samples passing quality control were available for 16 patients for cell-free plasma miRNA, 12 for exosomal miRNA and 10 for digital spatial profiling (Supplementary Table S4). To identify biomarkers that reflect treatment response and related molecular changes, miRNA expression was analyzed in both plasma and tumor-derived exosomes. miRNAs are considered excellent biomarkers due to their small size, stability, and presence in bodily fluids. Comprehensive miRNA profiling using the nCounter platform revealed significant molecular rewiring following cabozantinib treatment. Across plasma and tumor-derived exosomes, nine differentially expressed miRNAs were identified (6 downregulated, 3 upregulated; FDR < 0.05). Strikingly, *miR-873-3p* emerged as the sole miRNA concordantly downregulated in both compartments (log2 fold change = −1.8, *p* = 0.008) (Figure 2A,B). This conserved suppression across biofluids positions *miR-873-3p* as a candidate non-invasive biomarker of cabozantinib exposure, potentially reflecting systemic modulation of cabozantinib’s anti-angiogenic activity. Given the small number of paired samples and the absence of an external validation cohort, this observation requires confirmation in independent, adequately sized studies before any biomarker role can be claimed.

#### 3.5.2. Discriminating Responders from Non-Responders

**Figure 3:**
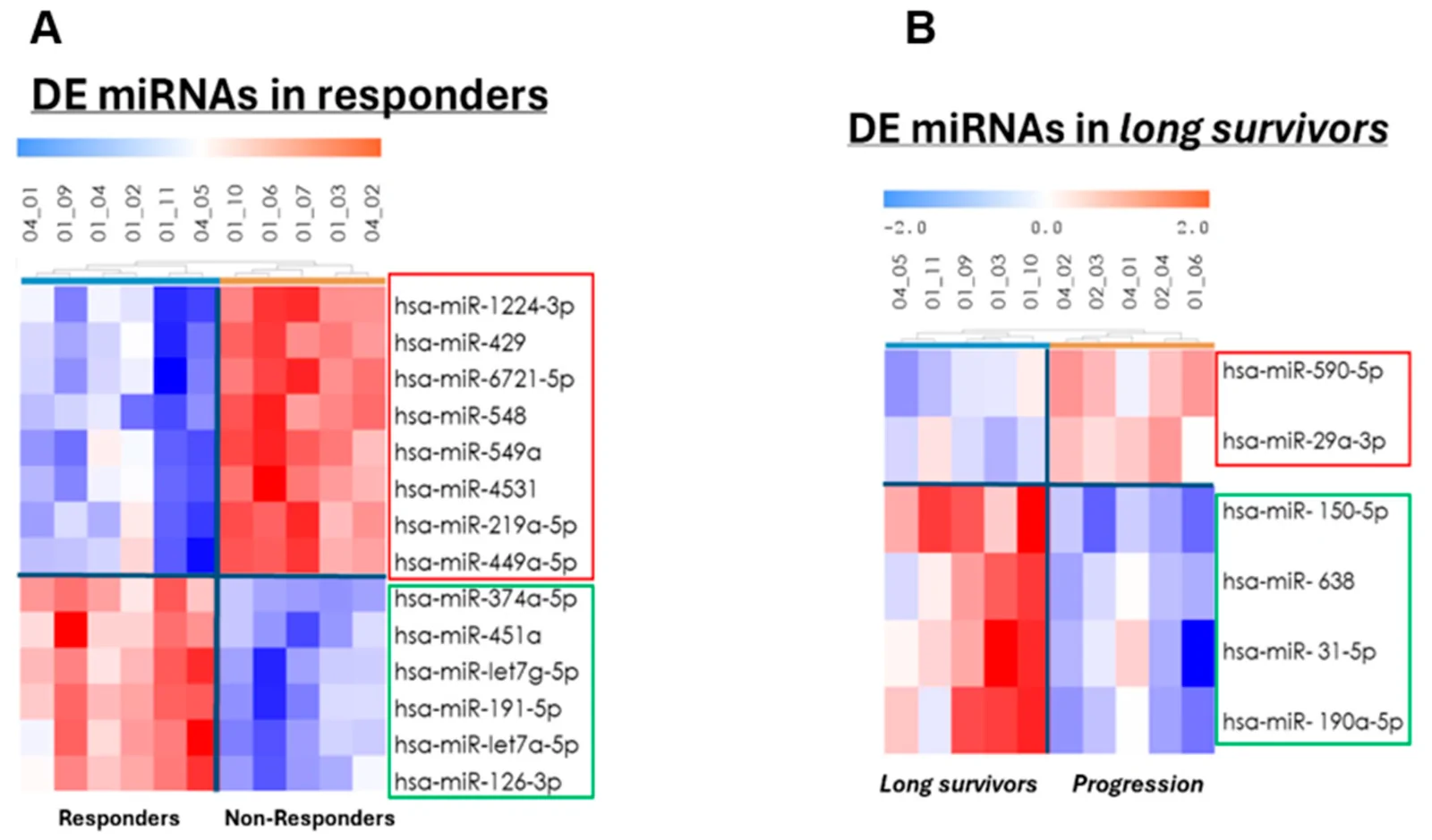
Impact of Cabozantinib on miRNA Expression Profiles in Plasma. (**A**) Heatmap showing the differential expression of miRNAs in molecular responders (≥10% tumor shrinkage) versus non-responders (<10%) after cabozantinib treatment. Each row represents a specific miRNA, and each column represents a sample. Red indicates higher expression and blue indicates lower expression. Selected miRNAs of interest are annotated on the right; green boxes highlight upregulated miRNAs in responders, while red boxes indicate downregulated miRNAs. (**B**) Heatmap showing the differential expression of miRNAs in long survivors versus patients with progressive disease after cabozantinib treatment. The layout and color scheme are consistent with Panel (A), allowing for a direct visual comparison of miRNA expression patterns. Selected miRNAs of interest are labeled on the right, with red boxes highlighting specific miRNAs for emphasis.

Comparative analysis between molecular responders (defined as patients with ≥10% tumor shrinkage) and non-responders identified a 14-miRNA signature comprising eight downregulated and six upregulated miRNAs (FDR < 0.05). Among the downregulated miRNAs, *miR-429* and *miR-1224-3p*—known suppressors of EMT and modulators of immune activation—were significantly reduced in responders, suggesting that their downregulation may facilitate epithelial–mesenchymal transition in this setting. Upregulated miRNAs included *miR-126-3p* (log_2_FC = 1.6, *p* = 0.01), a well-established regulator of VEGF signaling (Figure 3A).

#### 3.5.3. Descriptive Analysis of Extreme Responders

Given the limited sample size, we performed an exploratory descriptive analysis of extreme responders. In long-term responders (PFS > 12 months; *n* = 6), a distinct miRNA signature was identified (Figure 3B). Downregulated miRNAs included *miR-590-5p* and *miR-29a-3p*; the downregulation of *miR-29a-3p* aligns with its established role in extracellular matrix remodeling and EMT pathways, reflecting cabozantinib-mediated modulation of these processes. Upregulated miRNAs included *miR-150-5p*, *miR-638*, *miR-31-5p*, and *miR-190a-5p*, which have been associated with immune modulation and angiogenesis inhibition, potentially contributing to the extended PFS observed in this subgroup. Due to the small subgroup size, these findings are strictly exploratory and intended for hypothesis generation rather than formal survival correlation.

#### 3.5.4. Spatial Transcriptomic Profiling

**Figure 4:**
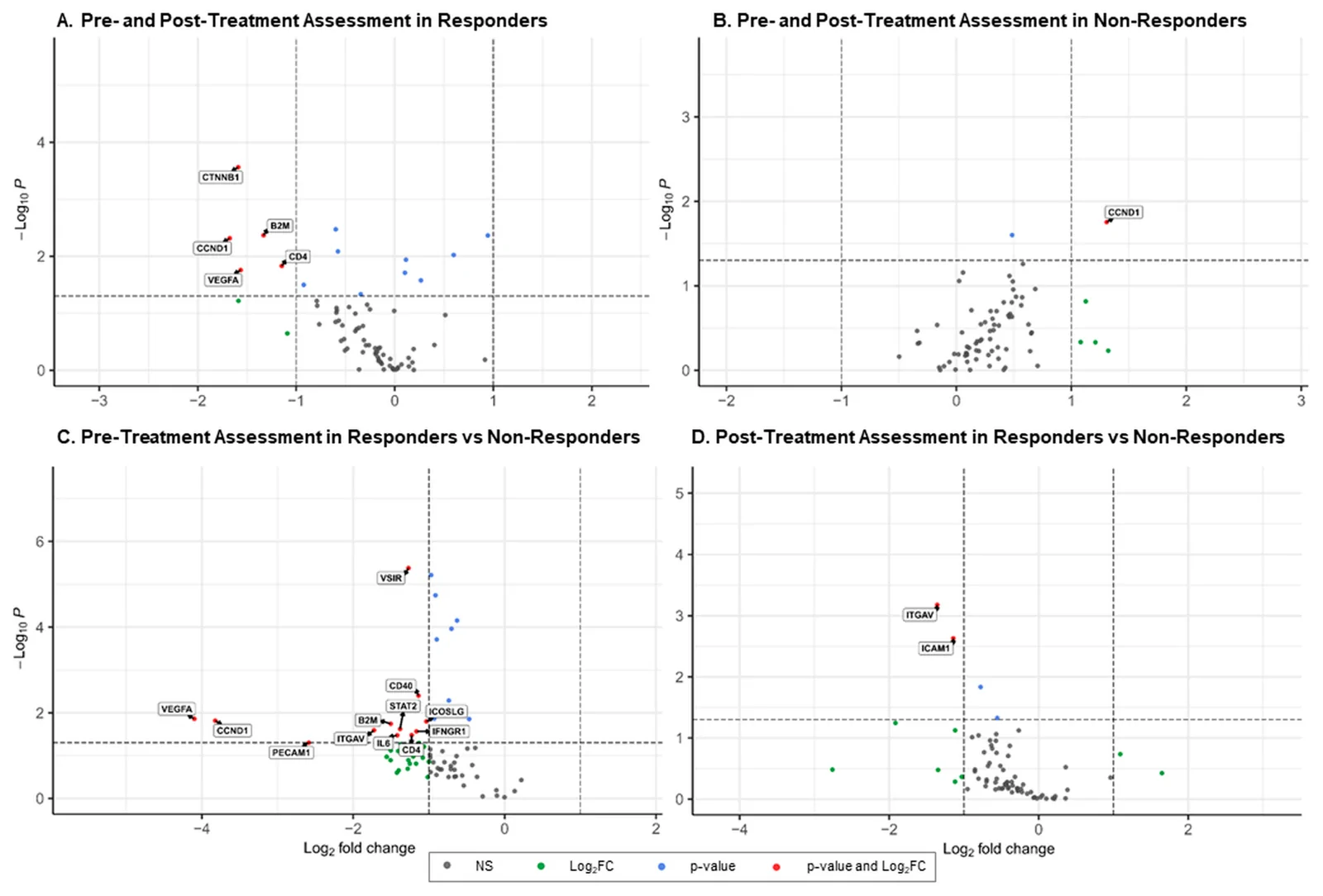
Digital Spatial Profiling: Gene Expression Patterns in Molecular Responders and Non-Responders to Cabozantinib. Volcano plot showing differential gene expression, with statistical significance versus fold change. The X-axis represents the log2 fold change in gene expression, and the Y-axis represents the -log10 of the unadjusted *p*-value. The dashed horizontal line marks *p* = 0.05 and the dashed vertical lines mark log2 fold changes of −1 and +1. Each point represents a gene, colored according to the default EnhancedVolcano scheme: grey, not significant; green, |log2 fold change| > 1 only; blue, *p* < 0.05 only; red, both. *p* values were not corrected for multiple testing, and all comparisons are nominal and exploratory Molecular Responders are defined as patients achieving ≥10% tumor shrinkage, while Non-Responders are those with <10% shrinkage. (**A**) Changes in gene expression in molecular Responders following treatment, based on comparisons between post-treatment and pre-treatment gene expression data. The volcano plot shows the decrease in expression of *VEGFA* and *CCND1* in responders after treatment with cabozantinib. *VEGFA* and *CCND1* are labeled to highlight their downregulation in responders after treatment. (**B**) Changes in gene expression in Non-Responders from pre- to post-treatment. The volcano plot shows the increase in expression of *CCND1* in non-responders after treatment with cabozantinib. *CCND1* is labeled to highlight its upregulation in non-responders. (**C**) Pre-treatment gene expression in non-responders compared to molecular Responders (**D**) Post-treatment gene expression in non-responders compared to molecular responders.

In this exploratory analysis, the proliferative and angiogenic markers *CCND1* and *VEGFA* were nominally downregulated after treatment in molecular responders (Figure 4A), whereas non-responders showed few changes in gene expression (Figure 4B). *ITGAV* was nominally upregulated in molecular responders before and after treatment, and *ICAM1* after treatment (Figure 4C,D). These changes are consistent with stromal remodeling. No direct vascular measurements were performed, so they cannot be interpreted as evidence of vascular normalization. *p* values are unadjusted and the comparisons involve few patients, so all findings are hypothesis-generating.

## 4. Discussion

The CABOPRE trial provides preliminary clinical evidence that perioperative cabozantinib is a feasible and active approach in patients with synchronous metastatic clear-cell RCC (ccRCC) eligible for deferred cytoreductive nephrectomy (CN). To our knowledge, this is the first prospective trial evaluating cabozantinib monotherapy specifically within this perioperative window, achieving substantial tumor downsizing with a manageable safety profile. Tumor shrinkage of any magnitude was observed in 86.6% of evaluable patients, enabling successful nephrectomy in 72.2% of the cohort. This surgical success rate is noteworthy given that the study population exclusively comprised patients with metastatic, bulky primary tumors, half of whom presented with poor-risk features, and 50% of patients with available staging data had clinical node-positive disease.

CABOPRE should be read against the evolution of cytoreductive nephrectomy over the past decade. CARMENA [9] showed that sunitinib alone was non-inferior to nephrectomy followed by sunitinib in patients with intermediate- and poor-risk disease, displacing upfront surgery from its position as a default intervention. SURTIME [6], closed early and underpowered for its primary endpoint, nonetheless suggested that deferring surgery until systemic response is documented may select patients who benefit, and its companion surgical analysis indicated that nephrectomy after sunitinib exposure is technically feasible without excess perioperative morbidity. CABOPRE was designed within that framework: cabozantinib was given not as definitive systemic therapy but as an induction phase intended to identify patients whose disease biology justifies surgery. Our observation that most patients reached nephrectomy with limited postoperative morbidity is consistent with the SURTIME safety experience, but the trial was not designed to test whether surgery confers benefit, and CARMENA remains the reference for that question.

Median overall survival in CABOPRE was 24.3 months. Without a control arm and with 18 patients, this estimate is imprecise, cannot be compared formally with historical series, and should not be read as evidence of a survival benefit from perioperative cabozantinib or from cytoreductive nephrectomy. Nonetheless, the clinical activity and low rate of treatment-limiting toxicities, with only one patient discontinuing therapy due to an adverse event, suggest that perioperative cabozantinib can be safely integrated into the multidisciplinary management of advanced RCC. This approach remains particularly relevant for selected patients in whom frontline immunotherapy-based regimens are contraindicated or unavailable [19].

The landscape of neoadjuvant therapy in RCC has evolved significantly. Historically, ORR for neoadjuvant targeted therapies ranged from 7% to 27% [14,20,21]. While contemporary frontline treatment has shifted toward ICI combinations (ICI-ICI or ICI-TKI) [22,23,24,25,26], investigating the specific contribution of VEGFR/MET/AXL inhibition remains mechanistically critical. Cabozantinib’s multi-targeted profile directly addresses tumor angiogenesis, a driving mechanism in ccRCC pathogenesis [27,28,29]. Our findings underscore its capacity to induce robust tumor cytoreduction, suggesting that optimized patient selection via predictive biomarkers could help identify candidates who may derive exceptional benefit from targeted TKI pathways.

The threshold used to define molecular responders warrants comment. Patients were dichotomised at 10% tumour shrinkage rather than the 30% required for a RECIST partial response. This was a pragmatic, post hoc decision: with only four confirmed partial responses, a RECIST-based dichotomy would have produced groups too small for comparison. The 10% cut-off separates patients with any measurable regression from those with none, and rests on the assumption that antiangiogenic effect may be detectable before it meets formal response criteria. This assumption is unverified. The threshold is arbitrary, was not prespecified, and may include patients whose tumour changes carry no biological meaning, attenuating true correlations. The molecular associations reported here should therefore be regarded as hypothesis-generating and require confirmation in cohorts large enough to use validated response definitions.

From a translational perspective, *miR-873-3p* was consistently downregulated post-treatment in both plasma and tumor-derived exosomes, highlighting its potential as a non-invasive liquid biopsy biomarker for monitoring therapeutic response [30]. Its suppression may modulate key microenvironmental processes; in other malignancies, alterations in this miRNA have been linked to chemoresistance, stemness, and regulation of angiogenesis (via EGLN3/HIF-1α), immune evasion (PD-L1), and cellular proliferation (FOXM1) [31]. Concordant downregulation across both systemic and tumor-derived compartments reinforces its utility for capturing systemic and tumor-specific molecular dynamics simultaneously.

Distinct miRNA expression patterns further differentiated molecular responders and long-term survivors from non-responders. The downregulation of *miR-429* and *miR-1224-3p*, alongside upregulation of *miR-126-3p*, a well-known regulator of VEGF signaling, in responders highlights modulation of EMT and angiogenic pathways, potentially reflecting tumor dependency on VEGFR and MET signaling. Similarly, long-term survivors exhibited increased expression of *miR-150-5p* and *miR-31-5p*, implicated in anti-angiogenic and hypoxia-regulating mechanisms, supporting their potential role as biomarkers for durable clinical benefit.

These microRNA findings were complemented by spatial transcriptomic data. In molecular responders, DSP demonstrated significant post-treatment downregulation of *VEGFA* and *CCND1*, a change consistent with anti-angiogenic and antiproliferative target engagement, whereas non-responders showed few transcriptional changes. The upregulation of *ITGAV* and *ICAM1* after treatment in these patients is compatible with stromal remodeling, aligning with recent spatial data linking COL4A1-ITGAV interactions with microenvironmental reprogramming and reinforcing the role of integrin αV as a potential biomarker and therapeutic target in ccRCC [32].

A primary limitation of this study is its early termination due to slow accrual and the rapid evolution of the first-line therapeutic standard toward ICI combinations. As a result, the study did not reach its full sample size, limiting statistical power and preventing formal subgroup analyses, including a definitive assessment of the survival impact of CN. The imprecision is substantial: with 15 evaluable patients, the 95% confidence interval for the objective response rate (7.8–55.1%) includes both the null (25%) and the alternative (45%) response rates of the Simon design. Survival comparisons by receipt of nephrectomy are further confounded by selection, because patients who progressed or deteriorated during induction could not undergo surgery, and by immortal time bias. All survival findings should therefore be considered exploratory. Surgical feasibility can be judged only from the rates of nephrectomy completion and Clavien–Dindo complications (Table S2). The translational analyses have their own limitations. No direct vascular measurements, such as vessel density, perfusion, pericyte coverage or hypoxia, were performed, so the changes in *VEGFA*, *ITGAV* and *ICAM1* cannot be interpreted as evidence of vascular normalization. Too few patients had both paired plasma and paired tumor samples to integrate the miRNA and spatial transcriptomic data within patients, and the two platforms are therefore reported separately. These constraints must be considered when interpreting the generalizability of our findings. Within-patient integration in larger cohorts with paired blood and tissue sampling, including on-treatment biopsies, is the priority for future work, together with independent validation of *miR-873-3p, ITGAV*, and *ICAM1*.

## 5. Conclusions

In CABOPRE perioperative cabozantinib was a feasible and tolerable in patients with synchronous metastatic ccRCC who were candidates for deferred cytoreductive nephrectomy, and 13 of 18 patients underwent surgery. The objective response rate (26.7%; 95% CI, 7.8–55.1) was imprecise and close to the 25% null rate, and the prespecified efficacy rule could not be applied because accrual stopped before the first stage of the Simon design was completed. Progression-free and overall survival, including all comparisons by IMDC risk group and by receipt of nephrectomy, are exploratory and do not support conclusions on the survival effect of cabozantinib or of cytoreductive nephrectomy. The circulating (*miR-873-3p*) and spatial (*ITGAV, ICAM1, VEGFA, CCND1*) markers identified here are hypothesis-generating; if validated in independent cohorts, they could inform the evaluation of cabozantinib as a component of neoadjuvant combination regimens.

## Figures and Tables

**Table 1:** Patient Characteristics (*n* = 18).

Characteristic	n (%)
Age (years)	Median (min, max) 57 (40–80.0)
Sex	
- Male	12 (66.7%)
- Female	6 (33.3%)
IMDC score	
- Intermediate	9 (50.0%)
- Poor	9 (50.0%)
ECOG PS	
- 0	4 (22.2%)
- 1	14 (77.8%)
Median primary tumor size, mm	96 mm (55–145)
Clinical T stage, n (%)	
- cT1	1 (5.6%)
- cT2	2 (11.1%)
- cT3	7 (38.9%)
- cT4	8 (44.4%)
Clinical N stage, n (%)	
- Missing data	4
- N0	7 (50.0%)
- N1	7 (50.0%)
Clinical M stage, n (%)	
- M1	18 (100.0%)
≥2 measurable metastatic sites, n (%)	12 (66.7%)
Location of metastases	
Lung	13 (72.2%)
Bone	7 (38.9%)
Liver	2 (11.1)

## Data Availability

The clinical trial protocol is available as Supplementary Material. The datasets generated and/or analyzed during the current study (including de-identified clinical characteristics, efficacy outcomes, and filtered plasma miRNA and Digital Spatial Profiling expression matrices) are not publicly available due to patient confidentiality and institutional privacy restrictions. In accordance with ICMJE guidelines, individual de-identified participant data and the statistical analysis plan will be made available by the corresponding author upon reasonable request for non-commercial research and validation purposes. Requests will be evaluated by the authors, and access will be granted within 30 days of receipt, subject to a formal data use agreement.

## Supplementary Materials

The following supporting information can be downloaded at: https://www.mdpi.com/article/10.3390/cancers18193216/s1, Table S1: Adverse events of any cause (safety analysis set, *n* = 18); Table S2: Surgical outcomes (full analysis set, *n* = 18); Table S3: Exploratory associations between IMDC risk factors and survival (*n* = 18); Table S4: Patients and samples in the biomarker and spatial transcriptomic analyses; Figure S1: Flow of patients and samples through the CABOPRE trial. File S1 Clinical Trial Protocol.

## Abbreviations

The following abbreviations are used in this manuscript:

AE	Adverse Event
ccRCC	Clear-cell Renal Cell Carcinoma
CI	Confidence Interval
CN	Cytoreductive Nephrectomy
CR	Complete Response
DSP	Digital Spatial Profiling
ECOG	Eastern Cooperative Oncology Group
EMT	Epithelial–Mesenchymal Transition
ERCC	External RNA Controls Consortium
FDR	False Discovery Rate
FFPE	Formalin-Fixed, Paraffin-Embedded
HR	Hazard Ratio
ICAM1	Intercellular Adhesion Molecule 1
ICI	Immune Checkpoint Inhibitor
IMDC	International Metastatic Renal Cell Carcinoma Database Consortium
IO	Immuno-Oncology
IQR	Interquartile Range
ITGAV	Integrin Subunit Alpha V
mRCC	Metastatic Renal Cell Carcinoma
miRNA	MicroRNA
NCCN	National Comprehensive Cancer Network
ORR	Objective Response Rate
OS	Overall Survival
PD	Progressive Disease
PFS	Progression-Free Survival
PR	Partial Response
RECIST	Response Evaluation Criteria in Solid Tumors
ROI	Region of Interest
SD	Stable Disease
TKI	Tyrosine Kinase Inhibitor
TMA	Tissue Microarray
TME	Tumor Microenvironment
TRAE	Treatment-Related Adverse Event
VEGFA	Vascular Endothelial Growth Factor A
