# Supplementary material for: CABOPRE: Evaluating Perioperative Cabozantinib’s Efficacy and Biomarkers for Cytoreductive Nephrectomy in Advanced Renal Cell Carcinoma
Source: Cancers (Basel). 2026 Oct 5;18(19):3216. doi: 10.3390/cancers18193216 (PMC13664424; doi:10.3390/cancers18193216)

Supplementary Materials

CABOPRE: Evaluating Perioperative Cabozantinib's Efficacy and Biomarkers for Cytoreductive Nephrectomy in Advanced Renal Cell Carcinoma

Contents

Table S1. Adverse events of any cause (safety analysis set, n = 18)

Table S2. Surgical outcomes (full analysis set, n = 18)

Table S3. Exploratory associations between IMDC risk factors and survival (n = 18)

Table S4. Patients and samples in the biomarker and spatial transcriptomic analyses

Figure S1. Flow of patients and samples through the CABOPRE trial

Table S1. Adverse events of any cause (safety analysis set, n = 18)

Preferred term	Patients, n (%)	Events, n	Grade 1	Grade 2	Grade ≥3	Treatment-related events, n
Asthenia	12 (66.7)	31	19	9	3	23
Diarrhea	11 (61.1)	22	16	6	0	20
Hypertension	10 (55.6)	14	9	5	0	14
Mucosal inflammation (mucositis)	7 (38.9)	9	5	4	0	8
Nausea	7 (38.9)	13	8	5	0	10
Decreased appetite	6 (33.3)	10	4	4	2	4
Transaminases increased	6 (33.3)	8	6	1	1	7
Vomiting	4 (22.2)	8	5	3	0	6
Palmar-plantar erythrodysesthesia	4 (22.2)	6	4	2	0	3
Pyrexia	4 (22.2)	4	3	1	0	1
Aphonia	3 (16.7)	6	3	3	0	6
Stomatitis	3 (16.7)	5	4	1	0	0
Dysgeusia	3 (16.7)	5	4	1	0	5
Epistaxis	3 (16.7)	3	3	0	0	1
Hypothyroidism	3 (16.7)	3	2	1	0	3
Back pain	2 (11.1)	3	1	2	0	0
Abdominal pain	2 (11.1)	2	1	1	0	0
Dyspnea	2 (11.1)	2	0	1	1	0
Hypercalcemia	2 (11.1)	2	1	0	1	0
Renal impairment	2 (11.1)	2	1	1	0	1
Weight decreased	2 (11.1)	2	1	1	0	1

Table S2. Surgical outcomes

Variable	Value
Nephrectomy	
Cytoreductive nephrectomy performed, n (%)	13 (72.2)
At the planned surgical visit, n	11
Deferred, performed after the CSR data cut-off, n	2
Nephrectomy not performed, n (%)	5 (27.8)
Early disease progression, n	1
Insufficient downstaging or persistent surgical risk, n	4
Perioperative timing	
Time from last cabozantinib dose to surgery, days, median (range)	34 (27–45)
Time from surgery to cabozantinib resumption, days, median (range)	21 (14–35)
Postoperative hospital stay, days, median (range)	5 (3–10)
Surgical complications (Clavien–Dindo)	
Patients with a surgical complication, n	1
Grade of the complication	IVa
Operative variables	
Surgical approach (open, laparoscopic, robotic)	Not collected
Operative time	Not collected
Estimated blood loss	Not collected
Warm ischemia time	Not collected

Table S3. Exploratory associations between IMDC risk factors and survival (n = 18)

Risk factor / variable	Median PFS, months	PFS HR (95% CI)	PFS p-value	Median OS, months	OS HR (95% CI)	OS p-value
Intermediate risk (1–2 factors) (reference)	16.3	1.00 (reference)	—	35.0	1.00 (reference)	—
Poor risk (≥3 factors)	2.9	4.25 (1.04–17.27)	0.043	7.7	1.33 (0.37–4.78)	0.665

This analysis was not prespecified and is presented as an unplanned exploratory analysis in response to a reviewer request. Hazard ratios are from univariable Cox proportional-hazards models; p-values are nominal and not adjusted for multiplicity. Given the sample size, the estimates are imprecise and are not intended for inference.

CI, confidence interval; HR, hazard ratio; IMDC, International Metastatic RCC Database Consortium; OS, overall survival; PFS, progression-free survival;

Table S4. Patients and samples in the biomarker and spatial transcriptomic analyses

Platform / analysis	Patients with samples, n	Excluded or failed quality control, n	Paired patients evaluated, n	Paired samples evaluated, n
Cell-free plasma miRNA (NanoString nCounter®)	18	2	16	32
Exosomal miRNA (NanoString nCounter®)	18	6	12	24
Digital spatial profiling (GeoMx® DSP)	12	2	10	20

Exclusions from the cell-free plasma miRNA analysis were due to low RNA quality or degradation; exclusions from the exosomal miRNA analysis were due to insufficient exosomal RNA concentration; exclusions from digital spatial profiling were due to absence of viable tumor tissue or failure of the renal core biopsy. The 20 paired samples evaluated by digital spatial profiling comprise 10 pre-treatment and 10 post-treatment specimens; the post-treatment specimens are 8 cytoreductive nephrectomy specimens and 2 renal core biopsies. DSP, digital spatial profiling; miRNA, microRNA.

Figure S1. Flow of patients and samples through the CABOPRE trial

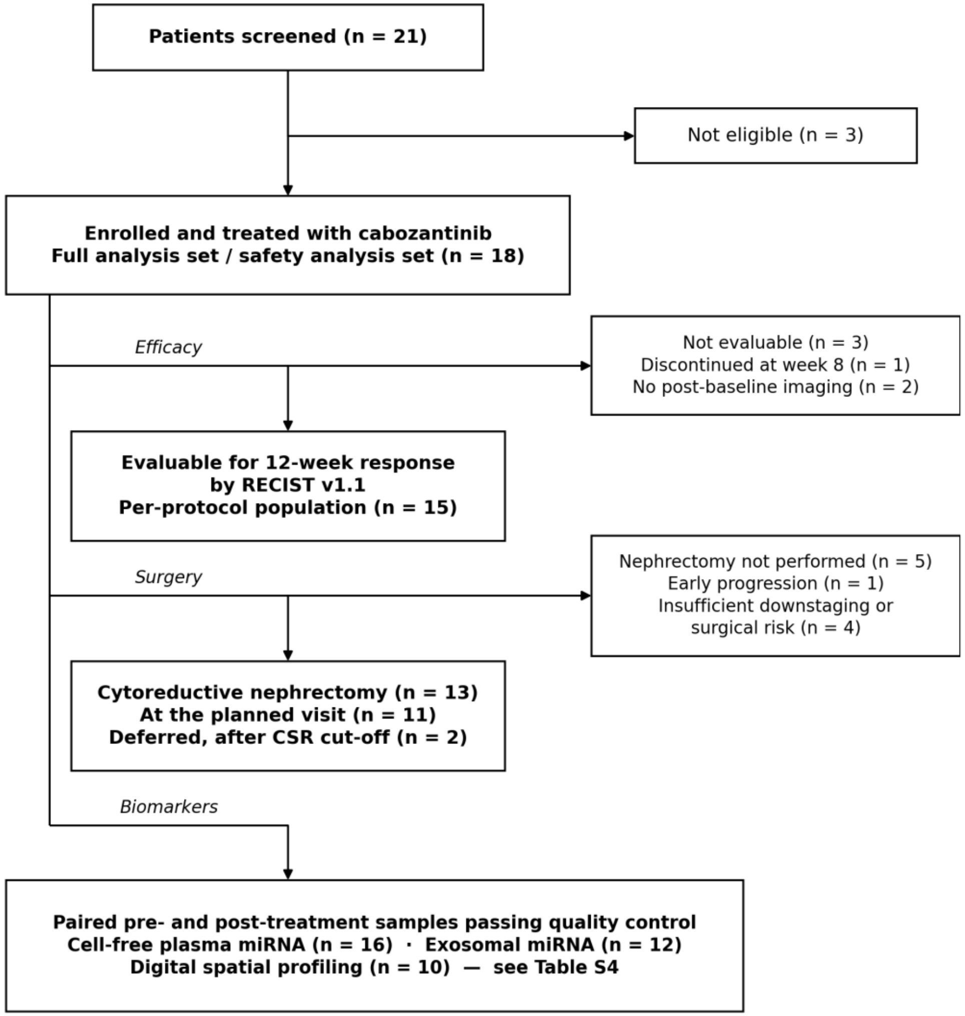

Supplement: Supplementary file 1 [file cancers-18-03216-s001.zip › cancers-4520292-supplementary.pdf]
