# Supplementary material for: CABOPRE: Evaluating Perioperative Cabozantinib’s Efficacy and Biomarkers for Cytoreductive Nephrectomy in Advanced Renal Cell Carcinoma
Source: Cancers (Basel). 2026 Oct 5;18(19):3216. doi: 10.3390/cancers18193216 (PMC13664424; doi:10.3390/cancers18193216)
Supplement: Supplementary file 1 [file cancers-18-03216-s001.zip › File S1. CABOPRE Clinical Trial Protocol.pdf]

Protocolo incluyendo Enmienda No Relevante nº 2

Estudio de fase II para la evaluación del tratamiento neoadyuvante con cabozantinib previo a la nefrectomía citorreductora en pacientes con cáncer de células renales localmente avanzado o metastásico

Código: CABOPRE

Versión 3.0 de fecha 14 de Octubre de 2019

EudraCT: 2018-001201-93

Protocolo incluyendo Enmienda nº 2 no relevante al protocolo	Versión 3.0	14 de Octubre de 2019
Protocolo incluyendo Enmienda nº 1	Versión 2.0	13 de Agosto de 2018
Protocolo	Versión 1.0	19 de Junio de 2018

Promotor:

FUNDACIÓN ONCOSUR

Gran Vía Marqués del Turia nº 65, 3º-11.

46005 Valencia

Correo electrónico: info@oncosur.org

Teléfono: 91 560 82 27

La información contenida en este documento es confidencial y no debe ser revelada a terceros salvo para obtener las autorizaciones previstas por la Ley.

FIRMAS INVESTIGADOR COORDINADOR Y REPRESENTANTE DEL PROMOTOR

Título: Estudio de fase II para la evaluación del tratamiento neoadyuvante con cabozantinib previo a la nefrectomía citorreductora en pacientes con cáncer de células renales localmente avanzado o metastásico

EudraCT: 2018-001201-93

Versión 3.0 de fecha 14 de Octubre de 2019

Declaran que el ensayo clínico será realizado en cumplimiento del protocolo, las Buenas Prácticas Clínicas y los requerimientos legales que apliquen.

Las modificaciones a este protocolo deberán presentarse previa conformidad del investigador coordinador / principal y del promotor.

Investigador Coordinator: Guillermo de Velasco, PhD, MD.

Firma y fecha: ______________________________

Promotor: *FUNDACIÓN ONCOSUR*

Nombre del representate: Dr. Luis Gonzaga Paz-Ares Rodríguez, Presidente

Firma y fecha: ______________________________

FIRMA DE CENTRO PARTICIPANTE E INVESTIGADOR PRINCIPAL

Centro:

Dirección:

Teléfono:

Fax:

Correo electrónico:

Yo, el abajo firmante, he revisado el protocolo titulado

" *Estudio de fase II para la evaluación del tratamiento neoadyuvante con cabozantinib previo a la nefrectomía citorreductora en pacientes con cán8er de células renales localmente avanzado o metastásico* ",

EudraCT: *2018-001201-93*

Versión 3.0 de fecha 14 de Octubre de 2019

y estoy de acuerdo en llevar a cabo este ensayo según el protocolo descrito en este documento, cumplir con sus requisitos, sin perjuicio de las consideraciones éticas y de seguridad y de acuerdo a lo establecido en este protocolo y conforme a la Declaración de Helsinki y la normativa aplicable sobre el uso de los medicamentos en investigación.

Investigador Principal:

Firma y fecha:

1 INFORMACIÓN GENERAL

1.1 TÍTULO

Estudio de fase II para la evaluación del tratamiento neoadyuvante con cabozantinib previo a la nefrectomía citorreductora en pacientes con cáncer de células renales localmente avanzado o metastásico

1.2 CÓDIGO

CABOPRE

1.3 VERSIÓN DEL PROTOCOLO Y FECHA

1.4 Versión 2.0 de fecha 13 de Agosto de 2018PROMOTOR

Persona autorizada por el promotor para firmar el protocolo y las enmiendas:

Dr. Luis Gonzaga Paz-Ares Rodríguez; Presidente

FUNDACIÓN ONCOSUR

Gran Vía Marqués del Turia nº 65, 3º-11.
46005 Valencia
Correo electrónico: info@oncosur.org
Teléfono: 91 560 82 27

1.5 MONITORIZACIÓN

Bioclever 2005 S.L.

Balmes 151, Primer Piso
Barcelona 08008
Teléfono: (34) 93 408 6388
Fax: (34) 93 467 2899
Info@bioclever.com

1.6 INVESTIGADOR COORDINADOR

Guillermo de Velasco

Hospital Universitario 12 de Octubre
Avda. Córdoba s/n
28041 Madrid
Teléfono: (34) 611482623
gdvelasco.gdv@gmail.com

1.7 CENTROS E INVESTIGADORES PARTICIPANTES

Ver ANEXO IV: Listado de centros e investigadores

ÍNDICE

1 INFORMACIÓN GENERAL 4
1.1 TÍTULO 4
1.2 CÓDIGO 4
1.3 VERSIÓN DEL PROTOCOLO Y FECHA 4
1.4 PROMOTOR 4
1.5 MONITORIZACIÓN 4
1.6 INVESTIGADOR COORDINADOR 4
1.7 CENTROS E INVESTIGADORES PARTICIPANTES 4
ABREVIATURAS 8
2 RESUMEN 9
3 ANTECEDENTES Y JUSTIFICACIÓN 14
3.1 CARCINOMA DE CÉLULAS RENALES 14
3.2 MET Y VEGFR-2 EN EL CARCINOMA DE CÉLULAS RENALES 14
3.3 CABOZANTINIB 15
3.3.1 Farmacología 15
3.3.2 Toxicología preclínica 15
3.3.3 Datos clínicos 15
3.3.4 Farmacocinética clínica de cabozantinib 16
3.4 CIRUGÍA CITORREDUCTORA 17
3.5 JUSTIFICACIÓN 18
3.5.1 Justificación del estudio de cabozantinib en el carcinoma de células renales 18
3.5.2 Justificación del diseño del estudio 18
3.5.3 Justificación del tiempo de dosis de cabozantinib preoperatorio 19
4 FINALIDAD Y OBJETIVOS DEL ESTUDIO 20
4.1 OBJETIVO PRINCIPAL 20
4.2 OBJETIVOS SECUNDARIOS 20
5 DISEÑO DEL ENSAYO 21
5.1 TIPO DE ENSAYO 21
5.2 CRITERIOS DE VALORACIÓN (ENDPOINTS) 21
5.2.1 Endpoint principal 21
5.2.2 Endpoints secundarios 21
5.2.3 Reclutamiento e inclusión de pacientes 22
5.3 CRITERIOS DE FINALIZACIÓN E INTERRUPCIÓN 22
5.4 VISITAS Y CRONOGRAMA 23
5.5 EVALUACIONES PREVISTAS 27
5.6 CALENDARIO PREVISTO 29

6 SELECCIÓN Y RETIRADA DE SUJETOS ... 29
6.1 CRITERIOS DE INCLUSIÓN ... 29
6.2 CRITERIOS DE EXCLUSIÓN ... 30
6.3 CRITERIOS DE RETIRADA ... 32
6.3.1 Retirada prematura de los pacientes ... 32
6.3.2 Actuación médica ante la retirada ... 32
6.3.3 Seguimiento después de una retirada prematura ... 32
6.3.4 Reemplazo de pacientes ... 32
7 TRATAMIENTOS ... 32
7.1 MEDICAMENTO EN INVESTIGACIÓN ... 32
7.1.1 Reducción de dosis ... 33
7.2 MEDICACIÓN CONCOMITANTE ... 34
7.2.1 Medicación prohibida ... 34
7.2.2 Medicación a utilizar con precaución ... 34
7.3 CUMPLIMIENTO TERAPÉUTICO ... 35
8 VARIABLES Y MEDIDAS ... 35
8.1 CARACTERIZACIÓN DE LA POBLACIÓN DE ESTUDIO ... 35
8.1.1 Características demográficas ... 35
8.1.2 Características clínicas ... 35
8.1.3 Parámetros de laboratorio ... 35
8.1.4 Características del cáncer renal ... 36
8.2 VALORACIÓN DE LA RESPUESTA ... 37
8.3 EVALUACIÓN DE LOS BIOMARCADORES ... 38
8.4 VALORACIÓN DE LA SEGURIDAD ... 38
9 ACONTECIMIENTOS ADVERSOS ... 38
9.1 DEFINICIÓN ... 38
9.2 CRITERIOS DE IMPUTABILIDAD ... 39
9.3 INTENSIDAD ... 40
9.4 PROCEDIMIENTO DE NOTIFICACIÓN DE ACONTECIMIENTOS ADVERSOS ... 40
9.4.1 Investigador ... 40
9.4.2 Promotor ... 41
9.5 HOSPITALIZACIÓN Y PROCEDIMIENTOS PREVISTOS ... 41
9.6 ALTERACIÓN DE UN PARÁMETRO DE LABORATORIO ... 42
9.7 DOCUMENTACIÓN RELACIONADA CON LOS AA Y AAG ... 42
9.8 SEGUIMIENTO DE LOS ACONTECIMIENTOS ADVERSOS ... 42
9.9 EMBARAZO ... 43
9.9.1 Varones ... 43
9.9.2 Consideraciones sobre fertilidad ... 43

10 ANÁLISIS ESTADÍSTICO 43
10.1 CONSIDERACIONES GENERALES 43
10.2 ANÁLISIS DESCRIPTIVOS 44
10.3 ANÁLISIS BIVARIANTE 44
10.4 EVALUACIÓN DE LA RESPUESTA (EFICACIA) 44
10.5 ANÁLISIS INTERMEDIOS 44
10.6 ANÁLISIS DE SEGURIDAD 45
10.7 CÁLCULO DEL TAMAÑO DE LA MUESTRA 45
10.7.1 Tamaño total de la muestra 45
10.8 DESVIACIÓN DEL PLAN DE ANÁLISIS ESTADÍSTICO 46
11 CONTROL Y GARANTÍA DE CALIDAD 47
11.1 MONITORIZACIÓN DEL ESTUDIO 47
11.2 AUDITORÍAS E INSPECCIONES 47
11.3 CUADERNO DE RECOGIDA DE DATOS 47
12 ASPECTOS ÉTICOS 49
12.1 CONSIDERACIONES GENERALES 49
12.2 INFORMACIÓN AL PACIENTE Y CONSENTIMIENTO INFORMADO 49
13 CONFIDENCIALIDAD Y PROTECCIÓN DE DATOS 50
13.1 ACCESO A LOS DATOS 50
13.2 CONFIDENCIALIDAD 50
13.3 PROTECCIÓN DE DATOS 50
13.4 ARCHIVO DE DOCUMENTACIÓN 51
13.4.1 Archivos del investigador y retención de documentos 51
13.4.2 Documentos fuente y datos básicos 51
14 FINANCIACIÓN Y SEGUROS 52
14.1 FUENTE DE FINANCIACIÓN 52
14.2 PÓLIZA DE SEGUROS 52
15 POLÍTICA DE PUBLICACIÓN 53
16 REFERENCIAS 54
ANEXO I: DEFINICIONES PARA EL ESTADIAJE DEL CÁNCER DE CÉLULAS RENALES DE ACUERDO CON LA CLASIFICACIÓN TNM DEL UICC 57
ANEXO II: ESCALAS DE VALORACIÓN FUNCIONAL 58
ANEXO III: CLASIFICACIÓN CLAVIEN-DINDO DE COMPLICACIONES QUIRÚRGICAS 59
ANEXO IV: LISTADO DE CENTROS E INVESTIGADORES 60

ABREVIATURAS

**AA**	Acontecimiento adverso
**AAG**	Acontecimiento adverso grave
**AEMPS**	Agencia Española de Medicamentos y Productos Sanitarios
**ALT**	Alanina aminotransferasa
**AST**	Aspartato aminotransferasa
**AUC**	Área bajo la curva
**BPC**	Buena práctica clínica
**CCAA**	Comunidades autónomas
**CCR**	Cáncer de células renales
**CEIm**	Comité ético de investigación con medicamentos
**CRDe**	Cuaderno de recogida de datos electrónico
**CRO**	Empresa de investigación por contrato
**EORTC**	Organización Europea para la Investigación y Tratamiento del Cáncer
**IC**	Intervalo de confianza
**IMDC**	*International Metastatic Renal Cell Carcinoma Database*
**IP**	Investigador principal
**INR**	Ratio internacional normalizada
**LDH**	Lactato deshidrogenasa
**LSN**	Límite superior de normalidad
**MSKCC**	*Memorial Sloan-Kettering Cancer Center*
**NC**	Nefrectomía citorreductora
**SG**	Supervivencia global
**SLP**	Supervivencia libre de progresión
**TC**	Tomografía computarizada
**TAC**	Tomografía axial computarizada
**TKI**	Inhibidor de la tirosina cinasa
**TTPa**	Tiempo de tromboplastina parcial activada
**VEGFR**	Factor de crecimiento endotelial vascular

2 RESUMEN

**Título**	Estudio de fase II para la evaluación del tratamiento neoadyuvante con cabozantinib previo a la nefrectomía citorreductora en pacientes con cáncer de células renales localmente avanzado o metastásico.
**Fase del estudio**	II
**Diseño**	Ensayo clínico de fase II, multicéntrico, nacional y no controlado para la evaluación del tratamiento con cabozantinib previo a la nefrectomía citorreductora.
**Población de estudio**	La población de estudio estará formada por pacientes con cáncer de células renales avanzado o metastásico candidatos a NC del tumor primario.<br>**Criterios de inclusión**<br>Los pacientes deben cumplir los siguientes criterios de inclusión:<br>1. Pacientes con cáncer de células renales localmente avanzado o metastásico con un componente de células claras confirmado histológicamente mediante biopsia.<br>2. Pacientes aptos para una NC de acuerdo con el riesgo quirúrgico establecido en la práctica clínica habitual del centro.<br>3. Edad ≥ 18 años.<br>4. Pacientes con un estado funcional ECOG de entre 0 y 1.<br>5. Pacientes con una función orgánica y de médula ósea adecuadas dentro de las 4 semanas previas al inicio del tratamiento con cabozantinib:<br>o Recuento de leucocitos > 4.000 células/µL<br>o Hemoglobina > 9 g/dL<br>o Recuento de plaquetas > 100.000/mm^3<br>o Creatinina sérica < 2,0 mg/dL o aclaramiento de la creatinina sérica > 30 mL/min (según la fórmula de Cockcroft-Gault (Cockcroft and Gault 1976)).<br>o Función hepática preservada: alanina aminotransferasa (ALT) y aspartato aminotransferasa (AST) < 2,5 x límite superior de la normalidad (LSN) en ausencia de metástasis hepáticas; < 5 x LSN en el caso de metástasis hepáticas.<br>o Lipasa < 2 x LSN; Amilasa < 2 x LSN .<br>o INR y TTPA ≤ 1,5 veces el LSN del centro. Esto solo aplica a pacientes que no reciban anticoagulación terapéutica; los pacientes que reciban anticoagulación terapéutica con heparinas de bajo peso molecular deben mantener dosis estables.<br>6. Pacientes sin evidencia radiológica o clínica de

pancreatitis.

7. Pacientes con enfermedad neoplásica evaluable según los criterios RECIST 1.1 (Eisenhauer et al. 2009).
8. Pacientes con tensión arterial controlada (sistólica < 145 mmHg; diastólica < 90 mmHg).
9. Pacientes que firman el consentimiento informado para participar en el estudio.
10. Los pacientes pueden tener metástasis cerebrales adecuadamente tratadas con radioterapia y/o cirugía (incluyendo radiocirugía), siempre y cuando no requieran el uso concomitante de corticoesteroides.

Criterios de exclusión

1. Pacientes con un segundo tumor maligno activo.
2. Pacientes con tumores tratados en los últimos 2 años.
3. Mujeres embarazadas o lactantes.
4. Mujeres en estado fértil que no acepten utilizar método contraceptivo durante el tratamiento. Las mujeres participantes en el estudio deberán haberse sometido a esterilización quirúrgica, estar en estado post-menopáusico o aceptar el uso de un método contraceptivo de alta eficacia (de acuerdo con los criterios del CTFG) durante el periodo de tratamiento. Tanto los pacientes de ambos sexos como sus parejas deben usar métodos anticonceptivos eficaces durante el tratamiento y, como mínimo, hasta cuatro meses después de completar el tratamiento. Dado que los anticonceptivos orales posiblemente no pueden considerarse “métodos anticonceptivos eficaces”, deben utilizarse junto con otro método, como un método de barrera.
5. Hombres en estado fértil que no acepten utilizar método contraceptivo durante el tratamiento. Los hombres participantes en el estudio deberán haberse sometido a esterilización quirúrgica o aceptar el uso de un método contraceptivo de alta eficacia (de acuerdo con los criterios del CTFG) durante el periodo de tratamiento.
6. Pacientes con alteraciones gastrointestinales, incluyendo:
    - Incapacidad para tomar medicación oral.
    - Necesidad de nutrición parenteral.
    - Procedimientos quirúrgicos previos que afecten la absorción.
    - Sangrado gastrointestinal activo.
    - Síndrome de malabsorción.
    - Alteraciones gastrointestinales que incrementen el riesgo de perforación.
7. Pacientes que hayan sufrido cualquiera de las siguientes condiciones dentro de los 12 meses previos a su inclusión en el estudio: infarto de miocardio, angina no controlada,

	hipertensión no controlada, bypass mediante injerto arterial periférico o coronario, insuficiencia cardiaca congestiva, accidente cerebrovascular o accidente isquémico transitorio.<br>8. Pacientes con tumores que invadan o afecten vasos sanguíneos mayores, el tracto gastrointestinal o la tráquea/bronquios.<br>9. Pacientes infectados con el virus de la inmunodeficiencia humana (VIH) o enfermedades relacionadas con el síndrome de inmunodeficiencia adquirida (SIDA).<br>10. Pacientes con hepatitis activa o con hepatitis C.<br>11. Pacientes con tuberculosis activa.<br>12. Pacientes con hipercalcemia no controlada (Ca > 12mg/dL) o sintomática que requiera tratamiento ininterrumpido con bifosfonatos o denosumab.<br>13. Pacientes sometidos a cirugía mayor dentro de las 4 semanas previas a la inclusión en el estudio.<br>14. Pacientes con hemorragias activas.<br>15. Pacientes con algún episodio reciente de obstrucción intestinal.<br>16. Pacientes con con alguna terapia previa dirigida al factor de crecimiento VEGF para enfermedad avanzada. .<br>17. Pacientes sometidos a radioterapia para las metástasis óseas durante las 2 semanas previas a la primera dosis de cabozantinib o sometidos a cualquier radioterapia externa dentro de las 4 semanas previas a la primera dosis de cabozantinib.<br>18. Pacientes que hayan recibido un trasplante alogénico o células madre en los últimos 5 años.<br>19. Neoplasias malignas en los 3 años anteriores al día 1 del ciclo 1, a excepción de los que tengan riesgo insignificante de metástasis o muerte y hayan sido tratados con intención curativa como el carcinoma in situ de cuello uterino o cáncer de próstata localizado tratado con intención curativa.<br>20. Pacientes que reciben un tratamiento anticoagulante a dosis terapéuticas con anticoagulantes orales (p. ej., warfarina, inhibidores directos de la trombina y del Factor Xa) o antiagregantes plaquetarios (p. ej., clopidogrel). El uso de heparinas de bajo peso molecular está permitido.<br>21. Hipersensibilidad al principio activo o a alguno de los excipientes (excluir a los pacientes intolerantes a la lactosa ya que cada comprimido recubierto con película contiene 46,61 mg de lactosa).<br>22. Cualquier otra condición clínica que considere el médico responsable del paciente como inadecuado para la inclusión en el estudio.

**Tamaño de la muestra**	– Número total de sujetos: 50<br>– Nº de sujetos en el estadio 1: 26<br>En caso de que más de 7 pacientes obtengan la respuesta objetiva durante el estadio 1, se procederá a reclutar el resto de pacientes.
**Objetivo principal**	– Evaluar la eficacia del tratamiento preoperatorio con cabozantinib, medida como la tasa de respuesta radiológica previa a la NC en pacientes con cáncer de células renales avanzado o metastásico candidatos a NC.
**Objetivos secundarios**	– Evaluar la tasa de respuesta radiológica durante el tratamiento con cabozantinib en pacientes con cáncer de células renales avanzado o metastásico candidatos a NC.<br>– Evaluar la supervivencia libre de progresión en pacientes sometidos a NC tras un tratamiento con cabozantinib.<br>– Evaluar la supervivencia global en pacientes sometidos a NC tras un tratamiento de 12 semanas con cabozantinib.<br>– Evaluar la seguridad y tolerabilidad de cabozantinib cuando se administra antes de la cirugía citorreductora, en base a la incidencia e intensidad de los acontecimientos adversos relacionados con el tratamiento notificados durante el estudio y clasificados según la CTCAE del NCI v4.03, así como las complicaciones quirúrgicas según la evaluación del sistema de clasificación de Clavien-Dindo.<br>– Evaluar los cambios en los marcadores moleculares séricos del cáncer de células renales asociados al tratamiento con cabozantinib en pacientes candidatos a una NC.<br>– Entender mejor el mecanismo de acción de cabozantinib.<br>– Evaluar la relación entre la expresión de C-MET tisular, C-MET en exosomas derivados del tumor y la eficacia del cabozantinib.<br>– Incrementar el conocimiento y la compresión de la biología de la enfermedad y la respuesta al fármaco. Para ello se realizarán los siguientes análisis (entre otros):<br>o Determinación del exoma tumoral, transcriptoma y proteoma.
**Fármaco de investigación**	Cabozantinib (Cabometyx®)<br>Grupo farmacoterapéutico: agentes antineoplásicos, inhibidores directos de la protein-cinasa, código ATC: L01XE26.
**Dosis y vía de administración**	Vía oral<br>60mg/día
**Duración del estudio**	Hasta progresión de la enfermedad o toxicidad intolerable. El tiempo máximo de seguimiento será de 12 meses (+/-1 mes) desde la entrada del último paciente en el estudio.

**Calendario previsto**	-Presentación a autoridades: 2º trimestre 2018<br>-Aprobación y apertura de centros: 3er trimestre 2018<br>-Primera visita del primer paciente: 4º trimestre 2018<br>-Inclusion último paciente: 4º trimestre 2019 (Periodo de inclusión 12 meses)<br>-Última visita del último paciente: 4ª trimestre 2020<br>-Cierre de Base de datos: 4º trimestre 2020<br>-Envío del informe final: 1er trimestre 2021

3 ANTECEDENTES Y JUSTIFICACIÓN

3.1 Carcinoma de células renales

El cáncer de células renales (CCR) comprende el 90% de carcinomas renales, y es considerado el 9º cáncer más frecuente en hombres y el 14º en mujeres (Eble 2004). En el año 2012, la incidencia global de cáncer de células renales fue de 338.000 casos, de los cuales 6.474 se diagnosticaron en España (Chavan et al. 2014). Si bien en la mayoría de países la mortalidad asociada al CCR se ha mantenido estable, se ha observado una tendencia al incremento en la incidencia de este cáncer (Znaor et al. 2015). Estas tendencias en la variación de la incidencia y la mortalidad, podrían ser atribuibles, por una parte, a la modificación de los principales factores de riesgo (tabaquismo y obesidad) y, por otra, al mayor diagnóstico accidental por el incremento en el uso de técnicas de imagen (Znaor et al. 2015).

El tratamiento de elección para el CCR localizado es la nefrectomía, ya sea con preservación o radical, dependiendo del tamaño del tumor primario (J Bellmunt et al. 2014). En el caso del CCR avanzado o metastásico, la inmunoterapia sistémica basada en citoquinas (especialmente la IL-2 y el TNFα) constituyó durante años el pilar del tratamiento farmacológico (Koneru and Hotte 2009). Estos agentes consiguieron superar la falta de eficacia observada con la quimioterapia, resultando en un incremento de la supervivencia en pacientes con CCR avanzado o metastásico. Sin embargo, a diferencia del tratamiento de otros tumores sólidos en estados avanzados, la extirpación quirúrgica del tumor primario se ha mantenido como tratamiento de elección en el CCR avanzado o metastásico (J Bellmunt et al. 2014; Joaquim Bellmunt et al. 2014). Además de ayudar al control de los síntomas, la resección quirúrgica del tumor primario previa al inicio de la inmunoterapia sistémica ha demostrado incrementar la supervivencia de los pacientes con CCR avanzado o metastásico (Flanigan et al. 2001; Mickisch et al. 2001).

En los últimos años, el mayor conocimiento sobre los mecanismos moleculares del CCR ha dado lugar al desarrollo de terapias dirigidas al bloqueo de las vías de señalización clave en el desarrollo del tumor, como el receptor del factor de crecimiento endotelial vascular (VEGFR) o la serina/treonina proteína cinasa mTOR. El CCR suele ser resistente a la quimioterapia y solo rara vez responde al tratamiento con hormonas o las citoquinas anteriormente mencionadas. Se ha demostrado que el tratamiento antiangiógeno tiene efectos clínicos beneficiosos en pacientes con CCR avanzado. La mayoría de estos fármacos nuevos han recibido la aprobación administrativa mediante el uso de la supervivencia sin progresión como marcador indirecto de efectos clínicos.

Actualmente, los fármacos de elección en esta terapia dirigida son los inhibidores de la tirosina cinasa sunitinib y pazopanib (J Bellmunt et al. 2014). Otros agentes con eficacia demostrada en la terapia dirigida incluyen inhibidores de la tirosina cinasa de VEGFR como axitinib y sorafenib, el inhibidor de mTOR everolimus y el inhibidor del punto de control PD-1 nivolumab (Motzer, Jonasch, et al. 2015; Motzer, Escudier, et al. 2015; Powles et al. 2016). La terapia dirigida ha permitido superar la era de las citoquinas en el tratamiento farmacológico del CCR avanzado o metastásico. Sin embargo, la eficacia de estas terapias en la segunda línea de tratamiento es limitada.

3.2 MET y VEGFR-2 en el carcinoma de células renales

La proteína del receptor del factor de crecimiento de los hepatocitos (MET) y el receptor 2 del VEGF (VEGFR-2) son tirosina cinasas con actividad de receptor que intervienen en el favorecimiento del crecimiento de las células tumorales, la capacidad de invasión, las metástasis o la angiogénesis en muchos tipos de tumores, incluido el CCR (Sierra and Tsao 2011). Los carcinomas renales de células claras tienen alterado el gen de Von Hippel-Lindau (VHL) (Choueiri et al. 2013), lo que desencadena un aumento de la expresión del VEGF y de la tirosina cinasa con actividad de receptor MET. Esto conduce a un aumento de

la angiogénesis y a un mayor crecimiento tumoral. La expresión elevada de MET se asociada a características anatomopatológicas desfavorables y a un peor pronóstico en el CCR (Kim, Kim, and Kim 2017).

Uno de los mecanismos de resistencia a tratamientos dirigidos contra VEGF es la regulación aumentada de la vía de señalización de MET. Por lo tanto, la inhibición combinada de VEGFR y MET presenta una mayor eficacia que la que se logra mediante la inhibición de cualquiera de estas vías por separado (Posadas et al. 2013).

3.3 Cabozantinib

3.3.1 Farmacología

Cabozantinib presenta una actividad inhibidora potente frente a varias tirosina cinasas con actividad de receptor que se sabe que influyen en el crecimiento tumoral, las metástasis y la angiogénesis. Las dianas principales de cabozantinib son MET, VEGFR2/KDR y RET con unos valores de CI50 (concentración asociada a una inhibición del 50%) en células de 8, 2 y 85 nM, respectivamente. Además, cabozantinib inhibe la fosforilación de KIT, FLT3 y AXL con unos valores de CI50 de 5, 11 y 42 nM, respectivamente.

Los datos procedentes de experimentos farmacodinámicos demuestran que cabozantinib inhibe MET y VEGFR2 *in vivo*. El tratamiento con cabozantinib produce efectos antiangiógenos en xenoinjertos tumorales, con una alteración de la vascularización que comienza en el plazo de 24 horas después de la administración, y sus efectos proapoptósicos asociados. En la ficha técnica y el manual del investigador se recoge un resumen de la farmacología de cabozantinib. Ambos documentos deben repasarse junto con este protocolo de investigación.

3.3.2 Toxicología preclínica

La toxicología preclínica de cabozantinib se ha definido en estudios de dosis únicas y repetidas realizados en diversas especies. Puede consultarse más información en la ficha técnica y el manual del investigador.

3.3.3 Datos clínicos

Cabozantinib es un fármaco de administración oral que actúa como inhibidor de las tirosina cinasas y que muestra afinidad no solo por VEGFR, sino también por otros receptores con un papel importante en el desarrollo del CCR, como c-MET y AXL (Yakes et al. 2011; Sierra and Tsao 2011). La eficacia y seguridad de cabozantinib en el tratamiento del CCR fue inicialmente evaluada en el ensayo clínico de fase 3 METEOR, en que cabozantinib se comparó con everolimus en pacientes con CCR avanzado que habían progresado tras un tratamiento con un inhibidor del VEGFR (Choueiri et al. 2015). La supervivencia libre de progresión (SLP) fue significativamente mayor en el grupo tratado con cabozantinib: mediana 7,4 meses (IC95% 5,6 – 9,1) vs 3,8 meses (IC95% 3,7 – 5,4) (p < 0,001). Los resultados favorables observados en la SLP se confirmaron en el análisis de supervivencia global (SG) con un seguimiento superior a 18 meses (Choueiri et al. 2016). La mediana de SG asociada al tratamiento con cabozantinib fue de 21,4 meses (IC95% 18,7 – no estimable) frente a los 16,5 meses (IC95% 14,7 – 18,8); p < 0,001). La respuesta objetiva fue del 17% (IC95% 13 – 22) con cabozantinib y 3% (IC95% 2 – 6) con everolimus. En el contexto de la primera línea de tratamiento, la eficacia y seguridad de cabozantinib fueron evaluadas en el ensayo clínico CABOSUN, en el que 157 pacientes con CCR metastásico fueron aleatorizados para ser tratados con cabozantinib o sunitinib (Choueiri et al. 2017). El tratamiento con cabozantinib incrementó la SLP de forma significativa: 8,2 meses (IC95% 6,2 – 8,8) frente 5,7 meses (3,4 – 8,1). La tasa de respuesta objetiva fue del 46% (IC95% 34 – 57) en los pacientes tratados con cabozantinib y 18% (IC95% 10 – 28) en los pacientes tratados con sunitinib.

3.3.4 Farmacocinética clínica de cabozantinib

El análisis de farmacocinética poblacional de cabozantinib se realizó con los datos recopilados en 289 sujetos tratados con cabozantinib con tumores sólidos. La dosis administrada fue de 140 mg/día por vía oral. La semivida efectiva prevista es de unas 55 horas, el volumen oral de distribución (V/F), de 349 l y el aclaramiento oral (CL/F) en estado de equilibrio, de 4,4 l/h. La semivida terminal (para predecir el lavado del fármaco) es de 120 horas aproximadamente. Tras la administración oral de cabozantinib, la mediana del tiempo hasta la concentración plasmática máxima (T_{max}) de cabozantinib osciló entre 2 y 5 horas después de la administración. La administración diaria repetida de cabozantinib en una dosis de 140 mg durante 19 días produjo una acumulación media de cabozantinib de 4-5 veces (a tenor del área bajo la curva de concentración plasmática del fármaco en función del tiempo [AUC]) en comparación con la administración de una dosis única; el estado de equilibrio se alcanzó el día 15. Cabozantinib presenta una unión elevada a las proteínas del plasma humano (≥ 99,7 %). Una comida rica en grasas aumentó los valores de C_{max} y AUC en un 41 % y 57 %, respectivamente, con respecto a la situación en ayunas en sujetos sanos que recibieron una dosis oral única de cabozantinib de 140 mg (estudio XL184-004).

Cabozantinib es un sustrato de la enzima CYP3A4 *in vitro*. La inhibición de dicha enzima redujo la formación del metabolito N-óxido XL184 en más de un 80 %. La inhibición de la enzima CYP2C9 tuvo un efecto mínimo sobre la formación de metabolitos de cabozantinib (es decir, una reducción < 20 %). La inhibición de las enzimas CYP1A2, CYP2A6, CYP2B6, CYP2C8, CYP2C19, CYP2D6 y CYP2E1 no afectó a la formación de metabolitos de cabozantinib. El AUC de cabozantinib aumentó un 38 % con la administración conjunta de ketoconazol, un inhibidor potente de la enzima CYP3A4, y un 77 % con la administración conjunta de rifampicina, un inductor potente de esa misma enzima.

Cabozantinib es un inhibidor no competitivo de la enzima CYP2C8 (K_{iap} = 4,6 μM), un inhibidor de tipo mixto de las enzimas CYP2C9 (K_{iap} = 10,4 μM) y CYP2C19 (K_{iap} = 28,8 μM) y un inhibidor competitivo débil de la enzima CYP3A4 (K_{iap} estimada = 282 μM) en preparaciones de microsomas hepáticos humanos (MHH). En los sistemas de análisis recombinante y en MHH se observaron valores de CI50 > 20 μM para las isoenzimas CYP1A2, CYP2D6 y CYP3A4. Sin embargo, los datos de un estudio clínico de interacciones farmacológicas en sujetos con tumores sólidos no revelaron un efecto acusado de las concentraciones de cabozantinib en estado de equilibrio (≥ 100 mg/día de EBL durante un mínimo de 21 días) sobre el AUC de rosiglitazona, un sustrato de la enzima CYP2C8, en administración conjunta. Por consiguiente, no cabe prever que cabozantinib inhiba notablemente la enzima CYP2C8 en la práctica clínica ni, por inferencia, otras isoenzimas CYP450 que mostraron una menor inhibición in vitro en comparación con la CYP2C8.

Cabozantinib es un inductor del ARNm de CYP1A1 en hepatocitos humanos incubados (es decir, 75%-100 % de inducción de ß-naftoflavona en el control positivo de CYP1A1), pero no del ARNm de CYP1A2, CYP2B6, CYP2C8, CYP2C9, CYP2C19 o CYP3A4 ni de actividades enzimáticas asociadas a isoenzimas.

Cabozantinib es un inhibidor (CI50 = 7,0 μM), pero no un sustrato, de las actividades de transporte de la gp-P en un sistema de análisis bidireccional con células MDR1-MDCK. Por consiguiente, cabozantinib tiene el potencial de aumentar las concentraciones plasmáticas de sustratos de la gp-P administrados conjuntamente.

En un estudio clínico de equilibrio de masas (estudio XL184-012) se recuperó aproximadamente el 81 % de la radiactividad total administrada durante un período de recogida de 48 días tras administrar una dosis única de 14C-cabozantinib a sujetos sanos (54 % en las heces y 27 % en la orina). Los resultados de un análisis de farmacocinética poblacional indicaron que la insuficiencia renal leve o moderada (aclaramiento de creatinina ≥ 30 mL/min) carece de un efecto clínicamente importante sobre el aclaramiento de cabozantinib.

Se dispone de datos preliminares correspondientes a los sujetos con insuficiencia hepática leve (clase A de Child-Pugh, n = 6) e insuficiencia hepática moderada (clase B de Child-Pugh, n=6) en comparación con sujetos sanos equiparables (sin insuficiencia hepática) (n=6). Los sujetos con insuficiencia hepática leve (clase A de Child-Pugh) tuvieron un AUC0-inf plasmática un 61% mayor con cabozantinib en comparación con sujetos sanos equiparables, mientras que los sujetos con insuficiencia hepática moderada (clase B de Child-Pugh) tuvieron un AUC0-inf plasmático un 67% mayor con cabozantinib en comparación con sujetos sanos equiparables. El t1/2,z terminal fue un 80% más largo en los grupos de clase A y B de Child-Pugh en comparación con sujetos sanos equiparables. Los valores de exposición (AUC0-últ y AUC0-inf), t1/2z y CL/f fueron similares en los grupos de clase A y B de Child-Pugh. La bioequivalencia de la formulación en comprimidos frente a la formulación en cápsulas se evaluó en un estudio de grupos cruzados (XL184-010).

Hay que señalar que cabozantinib se administra a diario y tiene una semivida plasmática aparente prolongada (aproximadamente 120 horas), una acumulación de aproximadamente 5 veces en el plasma en estado de equilibrio y una variabilidad interindividual en estado de equilibrio de la C_{max} moderadamente elevada (intervalo: CV de 34 % a 43 %). Por tanto, el aumento poco importante por encima del límite del IC superior de la C_{max} observado en este estudio de bioequivalencia de dosis únicas no representa una diferencia clínicamente significativa en cuanto a la farmacocinética plasmática en estado de equilibrio entre las formulaciones en comprimidos y cápsulas.

En un análisis de farmacocinética poblacional no se identificaron diferencias clínicamente relevantes en cuanto al aclaramiento de cabozantinib entre ambos sexos ni entre la raza blanca (89 %) y las demás razas (11 %). La farmacocinética de cabozantinib no se ve influida por la edad (20-86 años). En la ficha técnica y el manual del investigador se recogen más resultados de este y otros ensayos de farmacocinética clínica.

3.4 Cirugía citorreductora

En general, en la era de la terapia dirigida, la evidencia existente sobre la eficacia de una nefrectomía previa al tratamiento sistémico es limitada y se basa esencialmente en estudios retrospectivos (Chapin et al. 2011; Hanna et al. 2016; Heng et al. 2014). Las guías de práctica clínica reconocen el posible beneficio de esta estrategia en pacientes con un riesgo intermedio o con lesiones sintomáticas, si bien alertan de la ausencia de ensayos clínicos que respalden esta opción terapéutica (J Bellmunt et al. 2014). Aun así, se estima que 3 de cada 10 pacientes con CCR son sometidos a nefrectomía citorreductora previa al inicio de la terapia dirigida (Hanna et al. 2016). En los estudios METEOR y CABOSUN, el porcentaje de pacientes sometidos a nefrectomía citorreductora previamente al inicio del tratamiento con cabozantinib fue del 86% y 72%, respectivamente (Choueiri et al. 2016, 2017). El mayor inconveniente de llevar a cabo una nefrectomía citorreductora anterior al inicio de la terapia dirigida es el retraso significativo en el inicio del tratamiento farmacológico, que no puede iniciarse con garantías hasta que el paciente se ha recuperado de la intervención quirúrgica. Esta limitación es especialmente relevante en pacientes con una enfermedad metastásica agresiva o con un mal pronóstico, para los que este retraso puede comprometer severamente el control de la enfermedad. Además, es importante tener presente que, si bien la nefrectomía citorreductora tiene una tasa de complicaciones post-operatorias reducida, entre el 50% y el 60% de los pacientes podrían no llegar a recibir la terapia dirigida a tiempo (Hanna et al. 2016). Por otro lado, la frecuencia de complicaciones quirúrgicas asociadas a la nefrectomía citorreductora antes y después de la terapia dirigida es similar, lo que sugiere que hecho de haber recibido un ciclo de terapia dirigida sistémica no compromete el resultado de la cirugía (Chapin et al. 2011). El análisis retrospectivo de 35 pacientes que habían recibido sunitinib antes o después de la nefrectomía citorreductora mostró que si bien los pacientes que no respondían a sunitinib tenían un mal pronóstico, los que sí respondían a la terapia dirigida mostraban un mayor beneficio si habían recibido una primera línea de tratamiento antes de la nefrectomía (Stroup et al. 2013).

3.5 Justificación

3.5.1 Justificación del estudio de cabozantinib en el carcinoma de células renales

El papel de la nefrectomía citorreductora (NC) para pacientes con cáncer renal avanzado de células claras (ccRCC) que se presentan con una masa renal síncrona y metástasis no está bien definido. El tratamiento estándar actual es la NC seguida del tratamiento con el inhibidor de la tirosina cinasa (TKI) del factor de crecimiento endotelial vascular (VEGF). Hasta 2004, la CN tenía nivel de evidencia I dado que un análisis combinado de dos ensayos prospectivos aleatorizados de la Organización Europea para la Investigación y Tratamiento del Cáncer (EORTC) y SWOG demostró que la NC seguida de tratamiento con INF-α se asoció con un aumento general de 5,8 meses supervivencia global (SG) en comparación con la inmunoterapia sola (13,6 vs. 7,8 meses). La introducción de terapias dirigidas ha llevado a un enorme aumento en la supervivencia global en mRCC, lo que dificulta la interpretación del papel de NC (Sun et al. 2016). El impacto de CN en la SG en la terapia dirigida se está abordando con dos grandes ensayos clínicos en curso (CARMENA: NCT0093033 y SURTIME: NCT01099423). Los estudios que han examinado la NC en la era de la terapia dirigida sugieren que la NC puede ser beneficiosa en pacientes con mRCC (Hanna et al. 2016). El Consorcio Internacional de Base de Datos sobre el Carcinoma de Células Renales Metastásicas (n = 1658) examinó pacientes con metástasis sincrónicas para determinar el beneficio en términos de SG de pacientes tratados con terapias dirigidas que se sometieron a NC vs. no NC. La mediana de SG fue de 20,6 frente a 9,5 meses para los pacientes con y sin NC, respectivamente (CRI: 0,60; IC del 95%: 0,52-0,69; p <0,001) (Heng et al. 2014).

Un problema potencial con la secuencia de NC seguida de TKI es que hay un retraso significativo en iniciar la terapia dirigida a VEGF mientras los pacientes se recuperan de la nefrectomía. Esto es especialmente preocupante para los pacientes con enfermedad agresiva o características de pronóstico deficientes para quienes este intervalo puede ser desventajoso para el control de la enfermedad. Es importante destacar que, a pesar de la baja tasa de complicaciones postoperatorias mostradas en diferentes estudios, hasta 50-60% de los pacientes no reciben TKI a tiempo (Hanna et al. 2016). Aunque puede haber un aumento de complicaciones específicas relacionadas con la herida, las complicaciones quirúrgicas generales y el riesgo de complicaciones graves no son mayores después de la terapia dirigida prequirúrgica en comparación con la cirugía citorreductora inicial (Chapin et al. 2011).

Los pacientes que no responden al TKI primario tienen un pronóstico precario. Se ha demostrado que la NC combinada con TKI se asocia con un mejor resultado específico de la enfermedad en mRCC. Los respondedores a TKI primarios que se someten a NC pueden tener una mejor supervivencia específica de enfermedad y supervivencia global de los pacientes que se someten a NC primaria, seguido de TKI (Stroup et al. 2013). Cabozantinib ofrece una enfermedad controlada en casi el 80% de los pacientes (Choueiri et al. 2015) con ~ 20% de la tasa de respuesta objetiva en la segunda y tercera línea, que pronostica una respuesta más alta antes de la nefrectomía. Por lo tanto, el objetivo de este estudio es evaluar la eficacia, la seguridad y los cambios en los biomarcadores durante el tratamiento con cabozantinib en pacientes con mRCC antes del NC.

3.5.2 Justificación del diseño del estudio

A pesar de los resultados favorables con cabozantinib como primera línea de tratamiento en CCR avanzado o metastásico observados en el estudio CABOSUN (Choueiri et al. 2017), cabozantinib está actualmente indicado para el tratamiento de CCR avanzado en pacientes que han recibido una terapia previa dirigida al VEGFR. Por otro lado, los datos de eficacia de cabozantinib en un contexto perioperatorio provienen de las subpoblaciones con y sin nefrectomía previa incluidas en los ensayos clínicos aleatorizados, pero la eficacia del fármaco en este contexto no ha sido evaluada de forma específica. Por este motivo se ha diseñado un estudio de fase II para obtener información preliminar de eficacia.

El estudio ha sido concebido en dos estadios con el fin de garantizar únicamente su continuación en caso de que los resultados preliminares confirmen la evidencia clínica obtenida hasta el momento. De acuerdo con la respuesta objetiva observada en el estudio METEOR (cercana al 20%), se ha establecido un umbral de respuesta objetiva para la continuación del estudio del 25%, es decir, 7 de los 26 pacientes reclutados durante el estadio inicial.

3.5.3 Justificación del tiempo de dosis de cabozantinib preoperatorio

El presente estudio no ha sido diseñado con la finalidad de evaluar la eficacia comparada con aquellos pacientes que reciben el tratamiento de primera línea tras la cirugía del tumor primario. Por este motivo, se ha establecido un periodo de tratamiento previo a la nefrectomía citorreductora que se considere razonablemente corto para obtener una respuesta retardando lo mínimo posible la nefrectomía citorreductora.

El periodo de 4-12 semanas que habitualmente transcurre entre el diagnóstico y la nefrectomía ofrece una ventana de oportunidad para investigar la eficacia de tratamientos farmacológicos, administrados en un lapso de tiempo en que el paciente estaría esperando la cirugía. Actualmente se desconoce la duración óptima del tratamiento con cabozantinib administrado de forma preoperatoria, pero como tratamiento de primera línea se espera una mediana de tiempo hasta la respuesta de 1,9 meses. En este sentido, en caso de que el tratamiento sea eficaz, un periodo de 12 semanas (3 ciclos de 28 días) debería permitir observar una respuesta en la mayoría de pacientes.

Finalmente, es importante remarcar que el diseño del estudio también permitirá llevar a cabo un extenso análisis de biomarcadores en estos pacientes.

4 FINALIDAD Y OBJETIVOS DEL ESTUDIO

La finalidad del presente estudio es evaluar la eficacia y seguridad de la terapia neo-adyuvante con cabozantinib en pacientes con cáncer de células renales avanzado o metastásico previa a la resección del tumor primario mediante nefrectomía citorreductora (NC).

En base a este propósito general, se establecen los siguientes objetivos:

4.1 Objetivo principal

- Evaluar la eficaciadel tratamiento preoperatorio con cabozantinib, medida como la tasa de respuesta radiológica previa a la NC en pacientes con cáncer de células renales avanzado o metastásico candidatos a NC.

4.2 Objetivos secundarios

- Evaluar la tasa de respuesta radiológica durante el tratamiento con cabozantinib en pacientes con cáncer de células renales avanzado o metastásico candidatos a NC.
- Evaluar la supervivencia libre de progresión en pacientes sometidos a NC tras un tratamiento con cabozantinib.
- Evaluar la supervivencia global en pacientes sometidos a NC tras un tratamiento de 12 semanas con cabozantinib.
- Evaluar la seguridad y tolerabilidad de cabozantinib cuando se administra antes de la cirugía citorreductora, en base a la incidencia e intensidad de los acontecimientos adversos relacionados con el tratamiento notificados durante el estudio y clasificados según la CTCAE del NCI v4.03, así como las complicaciones quirúrgicas según la evaluación del sistema de clasificación de Clavien-Dindo.
- Evaluar los cambios en los marcadores moleculares séricos del cáncer de células renales asociados al tratamiento con cabozantinib en pacientes candidatos a una NC.
- *Entender mejor el mecanismo de acción de cabozantinib.
- *Evaluar la relación entre la expresión de C-MET tisular, C-MET en exosomas derivados del tumor y la eficacia del cabozantinib.
- *Incrementar el conocimiento y la compresión de la biología de la enfermedad y la respuesta al fármaco. Para ello se realizarán los siguientes análisis (entre otros):
  - Determinación del exoma tumoral, transcriptoma y proteoma.

* Nota: los tres últimos objetivos secundarios, orientados a una mejor comprensión del mecanismo del fármaco y la enfermedad, se completarán únicamente cuando exista disponibilidad de muestras y capacidad logística de los laboratorios para al macenar la muestra a -80ºC. En caso de que no se puedan obtener o conservar las muestras, no se considerará una desviación del protocolo. Los análisis se llevarán a cabo en el Centro Nacional de Investigaciones Oncológicas y en el Instituto de Investigación del Hospital 12 de Octubre. Los pacientes deberán otorgar su consentimiento a la conservación de muestras.

5 DISEÑO DEL ENSAYO

5.1 Tipo de ensayo

Ensayo clínico de fase II, multicéntrico, nacional y no controlado para la evaluación del tratamiento con cabozantinib previo a la NC.

La siguiente figura muestra el diseño general del estudio:

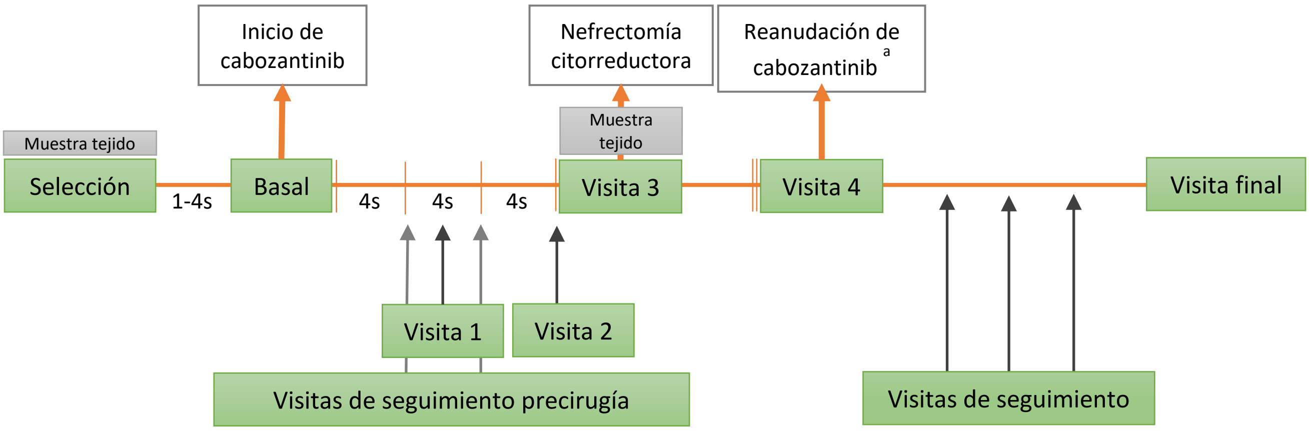

[a]El tratamiento con cabozantinib se reanudará cuando el paciente tenga una curación completa de la herida quirúrgica (a criterio del investigador) y hayan transcurrido un mínimo de 2 semanas desde la cirugía.

5.2 Criterios de valoración (endpoints)

5.2.1 Endpoint principal

- Respuesta objetiva al tratamiento preoperatorio con cabozantinib, definida como el porcentaje de pacientes que alcanzan una respuesta radiológica total o parcial a las 12 semanas de iniciar el tratamiento con cabozantinib, definidas de acuerdo con los criterios RECIST 1.1 (Eisenhauer et al. 2009) (ver sección 6. Variables y medidas).

5.2.2 Endpoints secundarios

- Mejor respuesta objetiva durante el tratamiento con cabozantinib, definida como el porcentaje de pacientes que alcanzan una respuesta radiológica total o parcial durante el tratamiento con cabozantinib.
- SLP, definida como el tiempo (en meses) transcurrido desde el inicio de tratamiento con cabozantinib hasta la progresión de la enfermedad (de acuerdo con los criterios RECIST 1.1. ver sección 6. Variables y medidas) o el fallecimiento del paciente.
- Porcentaje de pacientes libres de progresión (de acuerdo con los criterios RECIST 1.1) tras 12 meses del inicio del tratamiento con cabozantinib.
- SG, definida como el tiempo (en meses) transcurrido desde el inicio del tratamiento con cabozantinib hasta el fallecimiento del paciente.

- Frecuencia de acontecimientos adversos relacionados con cabozantinib y registrados durante el estudio.
- Variación de la concentración de los biomarcadores desde el inicio del tratamiento con cabozantinb hasta su finalización (el listado de marcadores a evaluar se describe en el apartado 8.3).

5.2.3 Reclutamiento e inclusión de pacientes

Para minimizar los sesgos en la selección de candidatos, el reclutamiento será consecutivo, ofreciendo la participación en el estudio de todos los pacientes con diagnóstico de cáncer renal metastásico en el momento del diagnóstico.

El estudio es abierto y no tiene grupo control, por lo que no se llevará a cabo ninguna aleatorización de los pacientes ni enmascaramiento del fármaco de estudio.

El periodo establecido para el cribado e inclusión de pacientes será de 12 meses.

El reclutamiento se llevará a cabo en dos estadios:

- **Estadio 1**. Se reclutarán un total de 26 pacientes. Para que el estudio pueda continuar, se requiere que más de 7 pacientes alcancen la respuesta objetiva.
- **Estadio 2**. En base a la tasa de respuesta objetiva obtenida en el análisis intermedio (llevado a cabo cuando el último paciente del primer estadio haya completado el objetivo principal), el investigador coordinador emitirá un dictámen en sobre la continuación o interrupción del estudio. En caso de que el informe sea favorable a la continuación del estudio, se procederá al reclutamiento del resto de pacientes, hasta llegar al tamaño de muestra de 50 pacientes.

Los investigadores principales (IP) deberán conservar un registro de todos los pacientes a los que se hayan realizado las pruebas de selección para participar en este estudio, incluidos los que no se consideren finalmente seleccionados para el estudio. Las copias de los registros de selección deberán guardarse en el archivo del investigador. Se deberá recoger el motivo principal de exclusión para cada paciente. Se pueden utilizar los datos de los estudios realizados como parte de la asistencia habitual del paciente para determinar la elegibilidad, siempre que se hayan obtenido dentro de los plazos definidos en el protocolo. Deberá obtenerse el consentimiento informado por escrito antes de la realización de cualquier procedimiento específico del estudio.

Después de asegurarse de que un paciente haya dado su consentimiento para participar en el estudio debe completarse el cuaderno de recogida de datos electrónico (CRDe). Se asignará a todos los pacientes una identificación única del ensayo para su uso en toda la correspondencia. Para garantizar la confidencialidad, los pacientes solo serán identificados mediante el identificador asignado del ensayo en el CRDe otros formularios específicos del estudio. Es responsabilidad del IP mantener la confidencialidad de los pacientes.

5.3 Criterios de finalización e interrupción

La fecha de final del ensayo será la última visita del último paciente.

En caso de que únicamente 7 o menos pacientes de los 26 reclutados en la primera fase alcancen la respuesta objetiva, el estudio será interrumpido definitivamente de acuerdo con el procedimiento descrito en el apartado 5.2.3).

Si el estudio se interrumpe prematuramente, deberán devolverse al promotor todos los materiales no utilizados. El IP se quedará con el archivo del investigador y una copia de los CRD completos.

5.4 Visitas y cronograma

Independientemente de las visitas de seguimiento que el centro programe de acuerdo a la práctica clínica habitual en pacientes tratados con cabozantinib, se llevarán a cabo las siguientes visitas:

- **Visita de selección** (entre 4 semanas y 1 día antes de iniciar el tratamiento). Los pacientes candidatos a participar en el estudio serán informados del mismo e invitados a participar. En caso de que el paciente acceda libremente a participar, firmará el correspondiente consentimiento informado, incluyendo el permiso para la obtención y preservación de muestras biológicas.

  Además de revisar los criterios de selección, para determinar si el paciente es apto para ser tratado con cabozantinib se extraerá una biopsia del tumor (o se recuperará una muestra de archivo del servicio de anatomía patológica, en caso de que la biopsia se hubiera llevado a cabo en el momento del diagnóstico). Si el paciente es apto (es decir, se confirma la localización exclusivamente renal del tumor primario y el examen de anatomía patológica revela un componente de células claras), se programará la primera visita de seguimiento pre-cirugía y es realizarán el resto de pruebas previstas. Si no es apto, se recogerán los motivos y el paciente será retirado del estudio. Si el paciente es una mujer, se realizará una prueba de embarazo. Además, en caso de que exista suficiente muestra y el paciente haya otorgado su consentimiento a la preservación, las muestras serán conservadas para su utilización en futuros estudios aprobados por un comité ético de investigación competente.

  Las pruebas previstas incluyen una una exploración física (con evaluación del estado funcional), una analítica de orina y sangre (incluyendo hemograma y bioquímica), una TC y un ECG. También se recogerá información sobre la medicación concomitante.

- **Visita Basal** (día 1 del primer ciclo de tratamiento con cabazanitib). En caso que se confirme el cumplimiento de todos los criterios de inclusión y ninguno de exclusión, no es necesario esperar 28 días desde la visita de selección para realizar al visita Basal y poder iniciar el tratamiento con cabozantinib. Se llevará a cabo una exploración física (con evaluación del estado funcional) y una analítica de orina y sangre (incluyendo hemograma y bioquímica). También se recogerá una muestra de plasma para el estudio de biomarcadores, que será preservada hasta el momento del análisis según se especifica en el manual de laboratorio. Se entregarán las unidades de cabozantinib necesarias para un ciclo de tratamiento y se recogerá información sobre la medicación concomitante.

- **Visitas de seguimiento pre-cirugía** (día 1 de los ciclos 2 y 3 de tratamiento pre-quirúrgico con cabozantinib). Se llevará a cabo una exploración física (incluyendo la evaluación del estado funcional) y una analítica de orina y de sangre, incluyendo hemograma y bioquímica. También se recogerá una muestra de plasma para el estudio de biomarcadores, que será preservada hasta el momento del análisis. Se entregarán las unidades de cabozantinib necesarias para un ciclo de tratamiento y se recogerá información sobre medicación concomitante y acontecimientos adversos registrados

- **Visita 1** (a las 6 semanas del inicio del tratamiento con cabozantinib). Se llevará a cabo una exploración física (incluyendo la evaluación del estado funcional) y una analítica de orina y de sangre, incluyendo hemograma y bioquímica. Se llevará a cabo un examen por tomografía computarizada (TC) de tórax, abdomen y pelvis (y ósea, sólo si clínicamente indicado) para valorar la respuesta al tratamiento y se recogerán y los acontecimientos adversos.

- **Visita 2** (a las 12 semanas +/- 1 semana del inicio del tratamiento con cabozantinib). Tras finalizar la primera fase de tratamiento con cabozantinib, se llevará a cabo una exploración física (incluyendo la evaluación del estado funcional), un ECG y una analítica de orina y de sangre, incluyendo hemograma y bioquímica. También se realizará una evaluación de la respuesta al tratamiento mediante TC.
- **Visita 3**. Nefrectomía citorreductora. Tras un mínimo de 28 días de la finalización del tercer ciclo de tratamiento con cabozantinib, se llevará a cabo la NC.

  Durante la NC se extraerá una muestra de tejido, que será preservada y enviada al laboratorio para una evaluación centralizada (el procedimiento de conservación y envio se encuentra detallado en el Manual de Laboratorio). En aquellos casos poco frecuentes en los que no es posible realizar una NC al final del tratamiento pre-cirugía con cabozantinib, se pedirá al paciente que done de manera opcional una muestra de tejido tumoral post-tratamiento, obtenida mediante biopsia por TC/ecografía, siempre que sea médicamente procedente. Las muestras de tumor obtenidas (ya sea durante la NC o mediante biopsia por TC-ecografía) serán analizadas en el propio centro. Además, en caso de que exista suficiente muestra y el paciente haya otorgado su consentimiento a la preservación, las muestras serán conservadas para su utilización en futuros estudios aprobados por un comité ético de investigación competente.
- **Visita 4**. Reanudación del tratamiento con cabozantinib. El tratamiento con cabozantinib se reanudará cuando el paciente tenga una curación completa de la herida quirúrgica (a criterio del investigador) y hayan transcurrido un mínimo de 2 semanas desde la cirugía. En caso de que el tratamiento se reanude transcurridas más de 8 semanas de la NC, se realizará una TC. Se entregarán las unidades de cabozantinib necesarias hasta la visita siguiente y se recogerá información sobre la medicación concomitante.
- **Visitas de seguimiento**. Las visitas de seguimiento se llevarán a cabo tras 12, 24 y 36 semanas (± 1 semana) de la NC. En estas visitas se llevará a cabo una exploración física (incluyendo la evaluación del estado funcional), un ECG y una analítica de orina y de sangre, incluyendo hemograma y bioquímica. Se llevará a cabo un examen por tomografía computarizada (TC) de tórax, abdomen y pelvis (y ósea, sólo si clínicamente indicado) para valorar la respuesta al tratamiento y se recogerán y los acontecimientos adversos.
- **Visita final**. Se llevará a cabo tras 12 meses de la entrada del paciente en el estudio o al finalizar el tratamiento por cualquier motivo.
- **Visita de seguimiento de seguridad**. La recogida de acontecimientos adversos se prolongará hasta un mes después del fin del tratamiento/estudio

En la siguiente página se muestra un cronograma con el detalle de las medidas llevadas a cabo en cada visita.

Cronograma del estudio

Procedimientos	Visita selección	Visita basal	V. de seguimiento pre-cirugía nº1	Visita 1	V.de seguimiento pre-cirugía nº2	Visita 2	Visita 3 NC	Visita 4	V. de seguimiento nº 1	V. de seguimiento nº 2	V. de seguimiento nº 3	V. Final	V. de seguimiento de seguridad
Día/Semana/Ciclo	*D-28 a D-1*	D1/C1	D1/C2<br>*+/- 3 días*	*6 semanas desde visita basal (inicio tratamiento) +/- 3 días*	D1/C3<br>*+/- 3 días*	*12+/- 1 semanas desde visita basal (inicio tratamiento)*	*mínimo 28 días de la finalización del 3er ciclo*	*mínimo 2 semanas desde la V3*<br>D1/C4	*12 +/-1 semanas desde V3*	*24 +/-1 semanas desde V3*	*36 +/-1 semanas desde V3*	*12 meses desde visita basal o fin de tratamiento*	*1 mes desde fin de tratamiento*
**Consentimiento**	x												
**Criterios de inclusión/exclusión**	x	x											
**Características clínicas del cáncer renal.**	x												
**Biopsia y evaluación histológica de tumor localmente[a]**	x						x						
**Muestra de biopsia para laboratorio central**	x						x						
**Datos demográficos y antecedentes médicos**	x												
**Exploración física [b]**	x	x	x	x	x	x		x	x	x	x	x	
**Estado funcional (ECOG) e índice de Karnofsky**	x	x	x	x	x	x		x	x	x	x	x	
**ECG [c]**	x					x			x	x	x	x	x
**Hematología y bioquímica en sangre [d]**	x	x	x	x	x	x			x	x	x	x	
**Análisis de orina[d]**	x	x	x	x	x	x			x	x	x	x	
**Proteinuria de 24 horas**				x		x		x	x	x	x	x	
**Prueba de embarazo[e]**	x	x	x		x			x	x	x	x		x
**Tomografía computarizada [f]**	x			x		x		x[j]	x	x	x	x	
**Valoración de la respuesta**				x		x			x	x	x	x	
**Muestras para Biomarcadores.**		x	x		x			x				x	
**Nefrectomía citorreductora**							x						
**Inicio de ciclo con cabozantinib**		x[h]	x[h]		x[h]			x[g,h]	x[h]	x[h]	x[h]		

Procedimientos	Visita selección	Visita basal	V. de seguimiento pre-cirugía nº1	Visita 1	V.de seguimiento pre-cirugía nº2	Visita 2	Visita 3 NC	Visita 4	V. de seguimiento nº 1	V. de seguimiento nº 2	V. de seguimiento nº 3	V. Final	V. de seguimiento de seguridad
Día/Semana/Ciclo	*D-28 a D-1*	D1/C1	D1/C2 *+/- 3 días*	*6 semanas desde visita basal (inicio tratamiento) +/- 3 días*	D1/C3 *+/- 3 días*	*12+/- 1 semanas desde visita basal (inicio tratamiento)*	*mínimo 28 días de la finalización del 3er ciclo*	*mínimo 2 semanas desde la V3* D1/C4	*12 +/-1 semanas desde V3*	*24 +/-1 semanas desde V3*	*36 +/-1 semanas desde V3*	*12 meses desde visita basal o fin de tratamiento*	*1 mes desde fin de tratamiento*
**Contabilidad de la medicación**			X		X	X			X	X	X	X	
**Medicación concomitante**		X	X	X	X	X	X	X	X	X	X	X	
**Recogida de acontecimientos adversos**	X	X	X	X	X	X	X	X	X	X	X	X	X[i]
**Fecha de finalización del tratamiento pre-cirugía.**							X					X	
**Fecha y motivo de finalización del tratamiento.**												X	
**Supervivencia**												X	X

a) En visita de selección se extraerá una biopsia del tumor (o se recuperará una muestra de archivo del servicio de anatomía patológica, en caso de que la biopsia se hubiera llevado a cabo en el momento del diagnóstico). En visita 3, en aquellos casos en los que los pacientes no son elegibles para una NC al final del tratamiento pre-cirugía con cabozantinib, se les informará y pedirá consentimiento para que donen una muestra de tejido tumoral post-tratamiento opcional, obtenida mediante biopsia por TC/ecografía, siempre que sea médicamente procedente.

b) Exploración física estándar debe incluir una determinación del peso (kg). La estatura (cm) se medirá solo en la visita de selección. Las constantes vitales incluirán la frecuencia respiratoria, la frecuencia cardíaca, la presión arterial sistólica y diastólica y la temperatura.

c) Realizados cada tres meses por riesgo de prolongación de QT durante el tratamiento, al final de estudio y cuando esté clínicamente indicado. ECG de 12 derivaciones (mediciones por triplicado).

d) Laboratorio local del centro. Los niveles de TSH se evaluarán en la visita de selección, a las 12 semanas des de la visita basal (visita 2) y luego cada 12 semanas desde Visita 3 hasta la visita final.

e) Prueba de embarazo en mujeres en edad fértil en suero u orina en la visita de selección y en orina antes de iniciar el tratamiento en cada ciclo y en la visita de seguridad.

f) TC de tórax , abdomen y pelvis (y ósea, sólo si clínicamente indicado). La TC de la visita de selección debe llevarse a cabo como máximo 4 semanas antes del inicio del tratamiento del estudio. Si la TC con contraste está contraindicada (p. ej., en pacientes con alergia al contraste o alteración del aclaramiento renal), se puede llevar a cabo mediante TC sin contraste o RMN. En la visita anterior a la NC debe utilizarse el método de evaluación del tumor usado en el periodo basal. Si el investigador lo considera oportuno, pueden repetirse en cualquier momento si se sospecha progresión de la enfermedad.(p. ej., TC o RMN).

g) El tratamiento con cabozantinib se reanudará cuando el paciente tenga una curación completa de la herida quirúrgica (a criterio del investigador) y tras un mínimo de 14 días post-cirugía

h) Entregar medicación suficiente hasta la siguiente visita de protocolo. Los ciclos duran 28 días (cada caja contiene 28 comprimidos). Antes de la cirugía el tratamiento será de 12 semanas (3 ciclos, C1, C2 y C3) ininterrumpidas. Luego, tras la cirugía en V4 se reanudará el tratamiento con un nuevo ciclo (C4), a continuación el tratamiento seguirá con nuevos ciclos (C5, C6, etc;) de manera ininterrumpida hasta la visita final (fin de estudio, progresión o toxicidad).

i) Cierre de los Aas que en la visita fin de estudio estuvieran abiertos.

j) La TC se realizará únicamente en caso de que el tratamiento se reanude más de 8 semanas después de la NC.

5.5 Evaluaciones previstas

Datos demográficos y antecedentes médicos

Se obtendrán los datos demográficos listados en el apartado 8.1, así como los antecedentes médicos relevantes, incluyendo los detalles de enfermedades de interés antes del consentimiento, intervenciones quirúrgicas, antecedentes oncológicos y antecedentes de tabaquismo. También se recogerá información acerca del diagnóstico del cáncer renal.

Exploración física y estado funcional.

Una exploración física estándar debe incluir una determinación del peso (kg) y debe llevarse a cabo en los momentos establecidos en la tabla de evaluaciones. La estatura (cm) se medirá solo en la visita de selección. Los resultados deben registrarse en la historia clínica como normales o clínicamente anómalos y debe proporcionarse información sobre los resultados clínicos anómalos.

Las constantes vitales incluirán la frecuencia respiratoria, la frecuencia cardíaca, la presión arterial sistólica y diastólica y la temperatura.

El estado funcional se evaluará en cada visita utilizando la puntuación del estado funcional ECOG e índice de Karnofsky, según lo especificado en el anexo II y se registrará en la historia clínica.

Electrocardiograma

En la selección, y cada tres meses durante y al final del tratamiento con cabozantinib y por seguridad (riesgo prolongación QT). Se obtendrán las lecturas del ECG de 12 derivaciones (mediciones por triplicado). En caso que esté clínicamente indicado se pueden realizar con más frecuencia. Debe registrarse en la historia clínica cualquier anomalía significativa del ECG.

Hematología, bioquímica, coagulación y análisis de orina

Las muestras de sangre para hematología, bioquímica clínica y pruebas de coagulación se obtendrán en la visita de selección y en las visitas que se describen en la tabla de evaluaciones, y se analizará en el laboratorio local del centro utilizando los métodos habituales empleados para la realización de análisis habituales. Se medirán las variables descritas en el apartado 8.1.3.Los valores analíticos que se considere que son de relevancia clínica se tienen que consignar como acontecimiento adverso (AA), salvo que la alteración del valor analítico esté relacionado con un AA ya registrado (p. ej., elevación de AST en una hepatitis). Se evaluarán los niveles de proteína en la orina (proteinuria de 24 horas) por el laboratorio local del centro, en Visita 1 (6 semanas), Visita 2 (12 semanas), Visita 4 (tras la cirugía), Visitas seguimiento 1,2,3 (12, 24 y 36 semanas) y en Visita final. A los pacientes con alteraciones analíticas clínicamente significativas se les debe realizar seguimiento hasta que los valores vuelvan a intervalos normales o hasta que se identifique una razón válida, que no sea un acontecimiento adverso relacionado con el fármaco.

Biomarcadores

Para el estudio de biomarcadores se obtendrán las muestras de sangre en las visitas detalladas en la tabla de evaluaciones siguiendo las instrucciones del manual de laboratorio.

Prueba de embarazo

Las pacientes con capacidad reproductiva deben obtener un resultado negativo en una prueba de embarazo en suero u orina entre 4 y 1 semana en la visita de selección, antes de cada ciclo y en la visita de seguridad en mujeres en edad fértil. Se debe documentar en la historia clínica la recomendación sobre anticoncepción.

Características clínicas y del tumor

Se recogerán los datos detallados en la sección 8.1.4

Evaluación histológica del tumor

Para la visita de selección, cuando existan muestras almacenadas, se solicitará permiso al paciente para analizar las muestras en el marco del estudio. En caso de que el paciente no disponga de ninguna muestra, se le realizará una biopsia. La muestra se procesará de acuerdo con lo descrito en el manual de laboratorio. Durante la NC se extraerá una muestra de tejido. En aquellos casos poco frecuentes en los que no es posible o aplicable la NC al final del tratamiento pre-cirugía con cabozantinib, se les pedirá que donen una muestra de tejido tumoral post-tratamiento de manera opcional, obtenida mediante biopsia por TC/ecografía, siempre que sea médicamente procedente. En caso de que exista suficiente muestra y el paciente haya otorgado su consentimiento a la preservación, las muestras de tumor obtenidas en la biopsia de la visita de selección y durante la NC serán conservadas para su utilización en futuros estudios aprobados por un comité ético de investigación competente.

Tomografía computarizada

Las evaluaciones del tumor se llevarán a cabo mediante TC (con contraste oral/i.v.) toracoabdominopélvica. Si la TC con contraste está contraindicada (p. ej., en pacientes con alergia al contraste o alteración del aclaramiento renal), se puede llevar a cabo mediante TAC sin contraste o RMN. Las evaluaciones del tumor deben realizarse como máximo 4 semanas antes del inicio del tratamiento del estudio y durante la visita anterior a la nefrectomía. En la visita anterior a la NC debe utilizarse el método de evaluación del tumor usado en el periodo basal (p. ej., TAC o RMN). Si el investigador lo considera oportuno, pueden repetirse TAC en cualquier momento si se sospecha progresión de la enfermedad.

Medicación concomitante

Todos los medicamentos (incluidos los medicamentos recetados y los adquiridos por el paciente sin receta) tomados por el paciente durante el periodo de selección, es decir, al menos 4 semanas antes del inicio de la administración de la medicación del estudio, se consignarán como medicación concomitante. Hay que tener en cuenta que debe registrarse toda la medicación para permitir la identificación clara de medicamentos con posibles interacciones con cabozantinib. Se recomienda evitar la prescripción de fármacos que puedan prolongar el intervalo QT.(Para más detalles sobre las medicaciones prohibidas y a utilizar con precaución ver punto 7.2)

Nefrectomía citorreductora (NC)

Debe llevarse a cabo una NC según la práctica habitual 12-16 semanas después de la inclusión en el estudio. Deberán haber transcurrido un mínimo de 28 días entre la

última dosis de cabozantinib y la cirugía. Los pacientes a los que no realice nefrectomía acudirán a visitas de seguimiento de seguridad.

En la historia clínica deben apuntarse fecha de la NC y resultado anatomopatológico. Se incluirá la evaluación del sistema de clasificación de Clavien-Dindo (Anexo III).

Entrega del fármaco

La medicación del estudio se entregará al paciente durante las visitas del día 1 de cada ciclo. Se registrará la fecha , el nombre del fármaco y la dosis del fármaco. Cuando se entregue el fármaco se revisará la ficha técnica y se realizará educación al paciente para identificar y minimizar (siempre que sea posible) los posibles acontecimientos adversos como el Síndrome de eritrodisestesia palmo-plantar o el Síndrome de leucoencefalopatía posterior reversible.

Registro de acontecimientos adversos

Se registrarán todos los acontecimientos adversos sucedidos desde la firma del consentimiento informado del estudio hasta un mes después del fin del tratamiento/estudio.

Con el fin de evaluar la seguridad y tolerabilidad de cabozantinib cuando se administra antes de la cirugía citorreductora se registrará la incidencia e intensidad según la CTCAE del NCI v4.03 de los acontecimientos adversos relacionados con el tratamiento. Se deberá prestar especial atención a los siguientes riesgos descritos con la utilización de cabozantinib: Síndrome de eritrodisestesia palmo-plantar y Síndrome de leucoencefalopatía posterior reversible.

5.6 Calendario previsto

- Presentación a autoridades: 2º trimestre 2018
- Aprobación y apertura de centros: 3er trimestre 2018
- Primera visita del primer paciente: 4º trimestre 2018
- Inclusion último paciente: 4º trimestre 2019 (Periodo de inclusión 12 meses)
- Última visita del último paciente: 4ª trimestre 2020
- Cierre de Base de datos: 4º trimestre 2020
- Envío del informe final: 1er trimestre 2021

6 SELECCIÓN Y RETIRADA DE SUJETOS

La población de estudio estará formada por pacientes con cáncer de células renales avanzado o metastásico candidatos a NC del tumor primario.

6.1 Criterios de inclusión

Los pacientes deben cumplir los siguientes criterios de inclusión:

1. Pacientes con cáncer de células renales localmente avanzado o metastásico con un componente de células claras confirmado histológicamente mediante biopsia.

2. Pacientes aptos para una NC de acuerdo con el riesgo quirúrgico establecido en la práctica clínica habitual del centro.
3. Edad ≥ 18 años.
4. Pacientes con un estado funcional ECOG de entre 0 y 1.
5. Pacientes con una función orgánica y de médula ósea adecuadas dentro de las 4 semanas previas al inicio del tratamiento con cabozantinib:
    - Recuento de leucocitos > 4.000 células/µL
    - Hemoglobina > 9 g/dL
    - Recuento de plaquetas > 100.000/mm^3
    - Creatinina sérica < 2,0 mg/dL o aclaramiento de la creatinina sérica > 30 mL/min (según la fórmula de Cockcroft-Gault (Cockcroft and Gault 1976)).
    - Función hepática preservada: alanina aminotransferasa (ALT) y aspartato aminotransferasa (AST) < 2,5 x límite superior de la normalidad (LSN) en ausencia de metástasis hepáticas; < 5 x LSN en el caso de metástasis hepáticas.
    - Lipasa < 2 x LSN, Amilasa < 2 x LSN.
    - INR y TTPA ≤ 1,5 vece el LSN del centro. Esto solo aplica a pacientes que no reciban anticoagulación terapéutica; los pacientes que reciban anticoagulación terapéutica con heparinas de bajo peso molecular deben mantener dosis estables.
6. Pacientes sin evidencia radiológica o clínica de pancreatitis.
7. Pacientes con enfermedad neoplásica evaluable según los criterios RECIST 1.1 (Eisenhauer et al. 2009).
8. Pacientes con tensión arterial controlada (sistólica < 145 mmHg; diastólica < 90 mmHg).
9. Pacientes que firman el consentimiento informado para participar en el estudio.
10. Los pacientes pueden tener metástasis cerebrales adecuadamente tratadas con radioterapia y/o cirugía (incluyendo radiocirugía), siempre y cuando no requieran el uso concomitante de corticoesteroides.

6.2 Criterios de exclusión

1. Pacientes con un segundo tumor maligno activo.
2. Pacientes con tumores tratados en los últimos 2 años.
3. Mujeres embarazadas o lactantes.
4. Mujeres en estado fértil que no acepten utilizar método contraceptivo durante el tratamiento. Las mujeres participantes en el estudio deberán haberse sometido a esterilización quirúrgica, estar en estado post-menopáusico o aceptar el uso de un método contraceptivo de alta eficacia [de acuerdo con los criterios del CTFG (CTFG 2014)] durante el periodo de tratamiento. Tanto los pacientes de ambos sexos como sus parejas deben usar métodos anticonceptivos eficaces durante el tratamiento y, como mínimo, hasta cuatro meses después de completar el tratamiento. Dado que los anticonceptivos orales posiblemente no pueden considerarse “métodos anticonceptivos eficaces”, deben utilizarse junto con otro método, como un método de barrera.
5. Hombres en estado fértil que no acepten utilizar método contraceptivo durante el tratamiento. Los hombres participantes en el estudio deberán haberse sometido a esterilización quirúrgica o aceptar el uso de un método contraceptivo de alta eficacia [de acuerdo con los criterios del CTFG (CTFG 2014)] durante el periodo de tratamiento.

6. Pacientes con alteraciones gastrointestinales, incluyendo:
   - Incapacidad para tomar medicación oral.
   - Necesidad de nutrición parenteral.
   - Procedimientos quirúrgicos previos que afecten la absorción.
   - Sangrado gastrointestinal activo.
   - Síndrome de malabsorción.
   - Alteraciones gastrointestinales que incrementen el riesgo de perforación.
7. Pacientes que hayan sufrido cualquiera de las siguientes condiciones dentro de los 12 meses previos a su inclusión en el estudio: infarto de miocardio, angina no controlada, hipertensión no controlada, bypass mediante injerto arterial periférico o coronario, insuficiencia cardiaca congestiva, accidente cerebrovascular o accidente isquémico transitorio.
8. Pacientes con tumores que invadan o afecten vasos sanguíneos mayores, el tracto gastrointestinal o la tráquea/bronquios.
9. Pacientes infectados con el virus de la inmunodeficiencia humana (VIH) o enfermedades relacionadas con el síndrome de inmunodeficiencia adquirida (SIDA).
10. Pacientes con hepatitis activa o con hepatitis C.
11. Pacientes con tuberculosis activa.
12. Pacientes con hipercalcemia no controlada (Ca > 12mg/dL) o sintomática que requiera tratamiento ininterrumpido con bifosfonatos o denosumab.
13. Pacientes sometidos a cirugía mayor dentro de las 4 semanas previas a la inclusión en el estudio.
14. Pacientes con hemorragias activas.
15. Pacientes con algún episodio reciente de obstrucción intestinal.
16. Pacientes con con alguna terapia previa dirigida al factor de crecimiento VEGF para enfermedad avanzada. .
17. Pacientes sometidos a radioterapia para las metástasis óseas durante las 2 semanas previas a la primera dosis de cabozantinib o sometidos a cualquier radioterapia externa dentro de las 4 semanas previas a la primera dosis de cabozantinib.
18. Pacientes que hayan recibido un trasplante alogénico o células madre en los últimos 5 años.
19. Neoplasias malignas en los 3 años anteriores al día 1 del ciclo 1, a excepción de los que tengan riesgo insignificante de metástasis o muerta y hayan sido tratados con intención curativa como el carcinoma in situ de cuello uterino o cáncer de próstata localizado tratado con intención curativa.
20. Pacientes que reciben un tratamiento anticoagulante a dosis terapéuticas con anticoagulantes orales (p. ej., warfarina, inhibidores directos de la trombina y del Factor Xa) o antiagregantes plaquetarios (p. ej., clopidogrel). El uso de heparinas de bajo peso molecular está permitido.
21. Hipersensibilidad al principio activo o a alguno de los excipientes (excluir a los pacientes intolerantes a la lactosa ya que cada comprimido recubierto con película contiene 46,61 mg de lactosa).
22. Cualquier otra condición clínica que considere el médico responsable del paciente como inadecuado para la inclusión en el estudio.

6.3 Criterios de retirada

6.3.1 Retirada prematura de los pacientes

Los pacientes finalizarán el ensayo clínico antes del tiempo estipulado en las siguientes circunstancias:

- Tras la biopsia realizada en la visita basal, el paciente es considerado no apto para recibir el tratamiento con cabozantinib.
- Progresión de la enfermedad metastásica durante el tratamiento con cabozantinib, de acuerdo con los criterios RECIST 1.1 (Eisenhauer et al. 2009).
- Interrupción del tratamiento durante más de 6 semanas debido a acontecimientos adversos.
- Mala adherencia al tratamiento, definida como menos del 70%, durante el estudio.
- Proceso o enfermedad intercurrente que en opinión del investigador requiera la retirada del paciente.
- Desviación del protocolo que en opinión del promotor requiera la retirada del paciente.
- Si la paciente es mujer y se queda embarazada.
- El paciente no desee continuar en el ensayo.

6.3.2 Actuación médica ante la retirada

En todos los casos se realizará la visita de finde estudio. Se aportará información detallada sobre la fecha y el motivo de interrupción. El investigador facilitará al paciente el soporte médico necesario.

6.3.3 Seguimiento después de una retirada prematura

Todos los pacientes que interrumpan prematuramente el estudio serán sometidos a un examen físico y a todas las pruebas especificadas en la visita de fin de estudio.

Al cabo de un mes del último dia de tratamiento recibido, se realizará una visita de seguimiento de seguridad.

6.3.4 Reemplazo de pacientes

Los pacientes retirados por desviación del protocolo en la inclusión de pacientes serán reemplazados por nuevos pacientes hasta llegar al número de pacientes previstos. Los pacientes retirados por otros motivos no serán reemplazados.

7 TRATAMIENTOS

7.1 Medicamento en investigación

Cabozantinib 60 mg

Denominación comercial: Cabometyx® 60 mg, comprimidos recubiertos con película. Cada comprimido recubierto con película contiene (S)-malato de cabozantinib, equivalente a 60mg de cabozantinib.

Código ATC: L01XE26

Régimen:

- Un comprimido diario durante 12 semanas.
- Cabozantinib es para administración por vía oral.
- Los comprimidos se deben tragar enteros y sin triturar. Se deben dar instrucciones a los pacientes para que no ingieran ningún alimento durante al menos 2 horas antes de tomar Cabozantinib y una hora después de hacerlo.
- La NC deberá llevarse a cabo tras un mínimo de 28 días de la finalización del tratamiento con cabozantinib.
- El tratamiento con cabozantinib se reanudará cuando el paciente tenga una curación completa de la herida quirúrgica (a criterio del investigador) y hayan transcurrido un mínimo de 2 semanas desde la cirugía.
- El paciente continuará con un comprimido diario de cabozantinib. En el caso que el paciente evolucione bien, el paciente seguirá con el mismo medicamento (salvo toxicidad o progresión).

Datos adicionales: ver– Manual del investigador/Ficha técnica

Cabozantinib será proporcionado gratuitamente por Ipsen Pharma durante el estudio cubriendo el tratamiento descrito en el protocolo des de visita Basal hasta la visita Final de cada paciente. El promotor será responsable de su distribución a los centros participantes una vez esté etiquetado y acondicionado.

Acondicionamiento, conservación y etiquetado

Todo el fármaco del estudio en investigación será suministrado por Ipsen Pharma al promotor, quien lo acondicionará y etiquetará conforme a la normativa local y el Anexo 13 de las Normas de Correcta Fabricación (AEMPS 2010). Se indicará que el fármaco es para uso exclusivo en ensayos clínicos y debe mantenerse fuera del alcance de los niños. La empresa ALCURA Healthcare, delegada por el promotor, será responsable de la distribución del fármaco a los centros investigadores; la gestión de la distribución del fármaco la realizarán conjuntamente el promotor y el equipo coordinador.

El IP de cada centro participante o una persona delegada (p. ej., un farmacéutico) será responsable de garantizar que el fármaco se almacene en un lugar seguro y en las condiciones de conservación recomendadas. El acceso debe limitarse al IP y los miembros autorizados del personal. El tratamiento del estudio debe mantenerse fuera del alcance de los niños. Este medicamento no requiere condiciones especiales de conservación (ver Sección 6.4 Precauciones especiales de conservación de la ficha técnica).

7.1.1 Reducción de dosis

De acuerdo con lo especificado en la ficha técnica de cabozantinib, en caso de que el investigador considere necesario reducir la dosis, se recomienda hacerlo a 40 mg diarios, y después a 20 mg diarios. Las reducciones de dosis serán irreversibles; es decir, una vez reducida la dosis en un determinado paciente, no se le volverá a subir.

El tratamiento con cabozantinib se debe interrumpir en pacientes que presenten Síndrome de leucoencefalopatía posterior reversible.

La siguiente tabla muestra las modificaciones de dosis según reacciones adversas tal y como se recomiendan en la ficha técnica:

Reacción adversa e intensidad	Modificación del tratamiento
Reacciones adversas de grado 1 y de grado 2 que sean tolerables y se puedan manejar fácilmente	Habitualmente no se precisa ningún ajuste de la dosis<br>Considerar complementar con medidas terapéuticas de apoyo según lo indicado.
Reacciones adversas de grado 2 que sean intolerables y que no se puedan tratar con una reducción de la dosis o medidas terapéuticas de apoyo.	Interrumpir el tratamiento hasta que la reacción adversa se resuelva hasta considerarse de grado ≤1.<br>Complementar con medidas terapéuticas de apoyo según lo indicado.<br>Considerar reiniciar el tratamiento con una dosis reducida.
Reacciones adversas de grado 3 (a excepción de anomalías de laboratorio sin relevancia clínica)	Interrumpir el tratamiento hasta que la reacción adversa se resuelva hasta considerarse de grado ≤1.<br>Complementar con medidas terapéuticas de apoyo según lo indicado.<br>Reiniciar el tratamiento con una dosis reducida.
Reacciones adversas de grado 4 (a excepción de anomalías de laboratorio sin relevancia clínica)	Interrumpir el tratamiento.<br>Instaurar medidas terapéuticas adecuadas.<br>Si la reacción adversa se resuelve hasta considerarse de grado ≤1, reiniciar el tratamiento con una dosis reducida.<br>Si la reacción adversa no se resuelve, interrumpir permanentemente la administración de CABOMETYX.

7.2 Medicación concomitante

El resto de tratamientos a parte del tratamiento con cabozantinib durante el ensayo se consideraran tratamientos concomitantes y se deben recoger en la historia clínica.

Se recordará a los pacientes participantes que no deben tomar tratamientos concomitantes sin el conocimiento y permiso del investigador.

En la Ficha técnica/Manual del investigador se detallan las interacciones farmacológicas descritas para cabozantinib.

7.2.1 Medicación prohibida

- Anticoagulantes orales (p. ej., warfarina, inhibidores directos de la trombina y del Factor Xa) y antiagregantes plaquetarios (aunque si se permite el uso de aspirina a dosis bajas).
- Terapia dirigida al factor de crecimiento VEGF

7.2.2 Medicación a utilizar con precaución

- Inhibidores de CYP3A4: Se debe proceder con precaución cuando cabozantinib se administre de forma conjunta con agentes que sean inhibidores potentes de CYP3A4 (p. ej., ketoconazol,ritonavir, itraconazol, eritromicina, claritromicina, zumo de pomelo)
- Inductores de CYP3A4: se debe evitar la administración crónica de agentes que sean inductores potentes de CYP3A4 con cabozantinib (p. ej., fenitoína, carbamazepina, rifampicina, fenobarbital o remedios naturales con hierba de San Juan [Hypericum perforatum])

- Sustratos de la glucoproteína P. Se debe advertir a los sujetos acerca del uso de sustratos de Pgp (p. ej., fexofenadina, aliskireno, ambrisentán, dabigatrán etexilato, digoxina, colchicina, maraviroc, posaconazol, ranolazina, saxagliptina, sitagliptina, talinolol, tolvaptán)
- Inhibidores de MRP2 se debe tener precaución en la administración concomitante de inhibidores de MRP2 (como ciclosporina, efavirenz o emtricitabina)
- Se recomienda evitar el uso de fármacos que puedan prolongar el QT (se debe utilizar con precaución en pacientes con antecedentes de prolongación del intervalo QT, pacientes que estén tomando medicamentos antiarrítmicos, o pacientes con enfermedad cardiaca preexistente relevante, bradicardia, o trastornos electrolíticos)
- Quelantes de las sales biliares

7.3 Cumplimiento terapéutico

El cumplimiento terapéutico se evaluará en base al recuento de la medicación de investigación. El servicio de farmacia del centro registrará el número de envases y comprimidos entregados al paciente. Tras la finalización del estudio, el paciente devolverá los envases no utilizados para el recuento de unidades. Se considerarán no adherentes al tratamiento aquellos pacientes que se hayan administrado menos del 70% de las dosis prescritas.

8 VARIABLES Y MEDIDAS

8.1 Caracterización de la población de estudio

Las siguientes características corresponderán al momento de la inclusión del paciente en el estudio.

8.1.1 Características demográficas

- Edad (años)
- Fecha de nacimiento
- Sexo

8.1.2 Características clínicas

- Índice de masa corporal (IMC) (Kg/m^2)
- Estado funcional del paciente
  - Puntuación de la escala **ECOG**. Escala de 0 a 5, en el que 0 significa que el paciente puede llevar a cabo una vida normal, sin limitaciones de ningún tipo y 5 la muerte (Anexo II).
  - Puntuación de la escala de **Karnofsky** (Puiggròs et al. 2009). Escala de 0 a 100 en incrementos de 10 puntos, en la que 100 indica ausencia de molestias o evidencia de la enfermedad y 0 la muerte (Anexo II)

8.1.3 Parámetros de laboratorio

Hematología (Aprox. 8 mL de sangre).

- Hemoglobina.
- Cifra de leucocitos con fórmula leucocitaria (neutrófilos, linfocitos).
- Cifra de plaquetas.

Bioquímica (Aprox. 6 mL de sangre)

- Glucosa.
- Urea.
- Creatinina.
- Sodio.
- Potasio.
- Magnesio.
- Cloruro.
- Bicarbonato.
- Calcio.
- Fósforo.
- Bilirrubina total
- ALT/AST.
- LDH.
- Albúmina.
- Amilasa
- Lipasa

Otras evaluaciones

- TSH

Pruebas de coagulación (Aprox. 4 mL de sangre)

- Tiempo de tromboplastina parcial activada (TTPa).
- INR

Análisis de orina

- Densidad relativa
- pH
- Glucosa
- Proteínas
- Cuerpos cetónicos
- Sangre
- Proteinuria 24 horas
- Prueba embarazo

8.1.4 Características del cáncer renal

- Fecha del diagnóstico del tumor primario.
- Fecha del diagnóstico de la enfermedad metastásica/avanzada.
- Fecha del inicio del tratamiento con cabozantinib
- Número de localizaciones metastásicas.
- Localización de las metástasis.
    - Hueso

  - Hígado
  - Cerebro
  - Otras (especificar)
- Estadío. Se recogerán los componentes *Tumor, Nodos y Metástasis* del sistema de estadificación T/N/M de 2009 (Sobin and Gospodarowicz 2009) (anexo I):
- Grupo de riesgo según el modelo de la *International Metastatic Renal Cell Carcinoma Database* (IMDC) (Ko et al. 2015):
  - Favorable
  - Intermedio
  - Alto

- Grupo de riesgo según el modelo del *Memorial Sloan-Kettering Cancer Center* (MSKCC/Motzer)(Motzer et al. 2002):
  - Riesgo favorable
  - Riesgo intermedio
  - Riesgo alto

8.2 Valoración de la respuesta

Para evaluar la respuesta de acuerdo a los criterios de valoración definidos en el apartado 5.2, se recogerán las siguientes variables en las visitas de seguimiento, visita 1, visita 2, visitas de seguimiento post-quirúrgico y visita final:

- Tipo de respuesta*, categorizada como:
  - **Respuesta completa**. Desaparición de todas lesiones neoplásicas y la reducción de los ganglios patológicos (si los hubiera) hasta un eje corto < 10 mm.
  - **Respuesta parcial**. Disminución de al menos el 30% en la suma de todos los diámetros de las lesiones neoplásicas, tomando como referencia la suma basal de los diámetros.
  - **Progresión**. Incremento de al menos un 20% en la suma de los diámetros de las lesiones neoplásicas, tomando como referencia la menor suma registrada en el estudio. Además, del incremento en porcentaje, la suma de los diámetros de las lesiones deberá incrementarse en 5 mm. La aparición de nuevas lesiones también será considerada progresión.
  - **Enfermedad estable**. No se cumplen los criterios para la respuesta parcial/total ni para la progresión, tomando como referencia la menor suma de diámetros registrada.

  *Las categorías del tipo de respuesta se han definido de acuerdo con los criterios RECIST 1.1 (Eisenhauer et al. 2009).
- Fecha de finalización del tratamiento
- Motivo de finalización del tratamiento:
  - Finalización del tratamiento previsto.
  - Progresión de la enfermedad metastásica (ver definición anterior).
  - Toxicidad asociada al tratamiento con cabozantinib.
  - Muerte.

- Otros (especificar).

8.3 Evaluación de los biomarcadores

Los siguientes parámetros serán evaluados tanto en la visita basal como en las visitas de seguimiento:

<u>Parámetros de laboratorio generales potenciales factores de riesgo:</u>

- Lactato deshidrogenasa (LDH)
- Calcio corregido (mg/dL)
- Hemoglobina (g/dL)
- Recuento de neutrófilos
- Recuento de plaquetas

<u>Biomarcadores específicos:</u>

- Concentración sérica del factor de crecimiento de los hepatocitos (pg/mL).
- Concentración sérica del factor de crecimiento vascular endotelial (pg/mL).
- Nivel de expresión del receptor c-Met:
  - 2
  - 2-4
  - > 4

8.4 Valoración de la seguridad

La seguridad y tolerabilidad del tratamiento con cabozantinib se valorará a partir del total de acontecimientos adversos registrados durante el seguimiento de los pacientes.

Además, se llevarán a cabo exámenes por tomografía computarizada de tórax, abdomen y pelvis (y hueso, cuando se considere apropiado) a las 6 semanas del tratamiento sistémico, antes de la nefrectomía (que tendrá lugar 12±1 semana después del inicio del tratamiento con cabozantinib) y cada 12 semanas tras la nefrectomía.

Dado que la NC es parte del diseño del estudio, esta intervención no será considerada un acontecimiento adverso, pero sí las complicaciones de la misma.

9 ACONTECIMIENTOS ADVERSOS

9.1 Definición

<u>**Acontecimiento adverso**</u> (AA) Evento médico presentado por un paciente o sujeto de investigación clínica al que se ha administrado un producto en investigación, pero que no necesariamente tenga una relación causal con el tratamiento.

<u>**Acontecimiento adverso grave**</u> (AAG) cualquier AA que, independientemente de la dosis:

- produce la muerte,
- pone en peligro la vida,
- precisa de ingreso hospitalario o lo prolonga,
- produce una discapacidad/incapacidad persistente o importante, así como una anomalía o defecto congénito,

- requiere una intervención para prevenir cualquiera de los resultados anteriormente mencionados, o
- se considera clínicamente relevante

Acontecimiento adverso inesperado (AAI): AA relacionado con el producto en investigación cuya naturaleza o intensidad no coincide con la información disponible con el producto administrado (Ficha técnica/Manual del investigador).

Acontecimiento adverso grave e inesperado (RAGI o SUSAR): AAG relacionado con el producto en investigación cuya naturaleza o intensidad no coincide con la información disponible con el producto administrado (Ficha técnica/Manual del investigador).

9.2 Criterios de imputabilidad

Ante un AA, el investigador deberá valorar el grado de relación con de causalidad con cabozantinib según el algoritmo del Sistema Español de Farmacovigilancia, en la que se definen las siguientes categorías de relación entre el AA y el fármaco en investigación:

Definitiva:

- Existe una secuencia temporal plausible en relación con la administración del fármaco o con los niveles plasmáticos o tisulares del mismo.
- La manifestación observada coincide con el esquema de reacciones adversas conocidas del fármaco implicado.
- No puede ser explicado por la enfermedad concurrente o por otros fármacos o sustancias químicas.
- La respuesta a la retirada debe ser clínicamente plausible. O sea mejora al interrumpir la administración del fármaco.
- El proceso de re-exposición debe ser positivo.

Probable:

- Existe una secuencia temporal razonable a la exposición del fármaco.
- La manifestación observada coincide con el esquema de reacciones adversas conocidas del fármaco implicado.
- Es poco probable que pueda atribuirse a la enfermedad intercurrente o a otros fármacos o sustancias químicas.
- Después de ser retirado el fármaco sigue una secuencia clínica razonable.
- Mejora al interrumpirse la administración del fármaco.
- No se requiere re-exposición para completar esta definición.

Posible:

- Existe una secuencia temporal razonable a la exposición del fármaco.
- Coincide con el esquema de reacciones adversas conocidas.
- Puede ser debida a la situación clínica del paciente o a otros fármacos o sustancias químicas administrados concomitantemente.
- La información sobre la retirada puede no existir o ser confusa.

Improbable:

- Un acontecimiento clínico, incluidas anomalías en las pruebas de laboratorio, con una relación temporal respecto a la administración del fármaco que hace improbable la relación de causalidad, y en el que otros fármacos, sustancias químicas o enfermedad intercurrente proporcionan explicaciones plausibles.

No relacionado:

No cumple ninguno de los criterios mencionados.

9.3 Intensidad

El investigador deberá evaluar la intensidad o magnitud de los AAs registrados, cuyo grado se clasificará de acuerdo con los criterios terminológicos comunes para los acontecimientos adversos (CTCAE) del *National Cancer Institute* (NCI), versión 4.03 (National Cancer Institute 2018):

Grado 1	Leve.
Grado 2	Moderado.
Grado 3	Intenso.
Grado 4	Potencialmente mortal.
Grado 5	Fatal, que causa la muerte.

9.4 Procedimiento de notificación de acontecimientos adversos

9.4.1 Investigador

El investigador comunicará al promotor de forma inmediata cualquier acontecimiento adverso grave y/o inesperado.

La comunicación debe realizarse dentro de las primeras 24 horas desde el inicio del acontecimiento adverso y se realizará usando el formulario de notificación.

Datos de contacto del promotor y el responsable de farmacovigilancia:

- **ONCOSUR**

  Fax: +34 91 460 33 10

  Teléfono: 91 560 82 27

  Correo electrónico: gdvelasco.gdv@gmail.com

- **Azierta**

  Teléfono: +34 912 771 076

  Fax: +34 911 982 394

  Correo electrónico: dfarmacovigilancia@azierta.eu

- **IPSEN PHARMA**

  Anna Roset Dalmau, Local Pharmacovigilance Manager

  Fax: +34 93 685 10 11

  Teléfono: +34 936858153 / +34 617052107

  Correo electrónico: pharmacovigilance.spain@ipsen.com, anna.roset@ipsen.com

Los acontecimientos adversos identificados durante el ensayo se recogerán en el CRD de cada participante).

Dependiendo de la naturaleza de la condición, cada AA se clasificará como:

- Grave / no grave
- Esperado / inesperado

El registro de los AAs es responsabilidad del equipo investigador, quien debe indicar el momento de aparición (expresado en la unidad más pequeña posible), su condición de grave / no grave y, en caso que se considere relacionado con el producto en investigación, si fue esperado o inesperado. Se deberá especificar la intensidad del evento (grado 1-5), las medidas adoptadas (ninguna, interrupción temporal o definitiva del producto en investigación), evolución (resuelto, resuelto con secuelas, persistencia) y la imputabilidad basándose en los criterios especificados en el apartado 9.2.

9.4.2 Promotor

La empresa AZIERTA, delegada por el promotor, notificará cualquier información importante de seguridad sobre el producto en investigación a la Agencia Española de los Medicamentos y Productos Sanitarios (AEMPS), las autoridades competentes de las comunidades autónomas (CCAA) y los comités de ética implicados en el ensayo clínico. Mantendrá en copia en las comunicaciones al proveidor del producto de investigación (IPSEN PHARMA).

AZIERTA notificará a la AEMPS todas las sospechas de reacciones graves e inesperadas (RAGI) asociadas al medicamento en investigación.

AZIERTA notificará a los órganos competentes de les CCAA todas las sospechas de RAGI asociadas al medicamento en investigación que hayan ocurrido en pacientes seleccionados en sus respectivos ámbitos territoriales.

A partir del momento que el promotor (o la empresa AZIERTA) haya tenido conocimiento de la sospecha de reacción adversa, el plazo de notificación será de:

- 15 días naturales.
- 7 días si el AAG e inesperado haya ocasionado la muerte o puesto en peligro la vida del sujeto. Más 8 días para completar toda la información.

Si la comunicación se realiza en formato electrónico, no será necesaria la notificación a las CCAA.

El promotor mantendrá un registro detallado de todos los AA comunicados por los investigadores.

Todos los AA serán notificados de forma tabulada en el informe final del estudio.

9.5 Hospitalización y procedimientos previstos

Si un sujeto tiene que ser hospitalizado o se le debe realizar algún procedimiento (por ejemplo, cirugía electiva) por cualquier evento o enfermedad previa a la inclusión y este está previsto antes de su inclusión en el ensayo (es decir, antes de la obtención del consentimiento informado), la hospitalización será considerada una intervención terapéutica y no como resultado de un AAG. Sin embargo, si el evento o la enfermedad empeoran durante la prueba, deberá notificarse como un AA (o AAG si el evento / enfermedad conlleva una situación grave como la hospitalización).

9.6 Alteración de un parámetro de laboratorio

Una alteración de un parámetro de laboratorio se considerará un AA si la anormalidad:

- resulta en la retirada del estudio,
- requiere tratamiento, modificación de dosis o interrupción del producto en investigación u otra intervención terapéutica, o
- es considerado clínicamente relevante

Independientemente de su intensidad, sólo las alteraciones de laboratorio relevantes que cumplan los criterios de gravedad serán registradas como AAG.

Si la alteración del laboratorio es parte de un diagnóstico o síndrome, sólo el síndrome o diagnóstico se incluirá como AA / AAG. Si la alteración de laboratorio no es parte de un diagnóstico o síndrome, se recogerá como AA o AAG.

Los cambios significativos en parámetros de seguridad que están asociados con la enfermedad en estudio no se consideraran como AA / AAG, a menos que el investigador crea que son más severos que los esperados dada la condición del paciente.

9.7 Documentación relacionada con los AA y AAG

Cada AA y AAG que ocurra durante el estudio debe ser documentado en la historia clínica del paciente, de acuerdo a la práctica clínica habitual del investigador, y en el CRD. Para cada AAG se usará un set independiente de formularios de AAG. Sólo si hay múltiples AAG al mismo tiempo y estos se interrelacionan temporal u clínicamente, se podrán registrar en un mismo formulario de AAG.

El investigador intentará realizar un diagnóstico del acontecimiento basándose en los signos, síntomas y/o otra información clínica. Se registrará un diagnóstico per línea o por signo/síntoma si el diagnóstico no está disponible. Si más adelante aparece un diagnóstico, se incluirá este y el signo/síntoma será tachado (con iniciales y fecha del investigador).

Las páginas de AAG que se encuentran en el archivo del investigador deben ser completadas de la forma más precisa posible y deben ser firmadas por el investigador antes de enviarlas al promotor. Es muy importante que en la página inicial el investigador indique su opinión sobre la relación con el producto en investigación.

9.8 Seguimiento de los acontecimientos adversos

El investigador debe recoger todos los AA ocurridos desde el momento que el paciente firma el consentimiento informado hasta 28 días después de la última dosis del producto en investigación.

Los AA se seguirán hasta 28 días después de la última dosis de cabozantinib.

Los AAG se seguirán preferiblemente hasta:

- resolución del acontecimiento,
- estabilización del acontecimiento, o
- vuelta a la condición basal del acontecimiento, en el caso que se disponga de condición basal.

En caso contrario, se seguirán hasta que:

- el evento pueda atribuirse a productos distintos al producto en investigación o factores no relacionados con el ensayo, o

- es poco probable obtener más información.

El investigador debe asegurar que los informes de seguimiento incluyan información adicional para permitir una evaluación completa de la naturaleza y/o causa/efecto del AAG, incluyendo pruebas de laboratorio, informes patológicos o visitas con especialistas.

9.9 Embarazo

Los casos de embarazo y sospechas de embarazo (incluidas las pruebas de embarazo con resultado positivo, con independencia de la edad o enfermedad) en una paciente en tratamiento con el producto en investigación o hasta 30 días después de su última dosis del producto en investigación se consideran eventos de comunicación inmediata y se registrarán como AA y sólo se registrarán como AAG si cumplen criterios de gravedad; deberán comunicarse inmediatamente al promotor mediante el formulario de notificación de AAG. La exposición al medicamento en investigación de cualquier mujer embarazada tiene que ser comunicada de forma inmediata también.

Si la paciente está tomando el producto en investigación, éste debe suspenderse inmediatamente y la paciente debe devolver todo el medicamento sin usar al investigador.

El investigador realizará un seguimiento de la paciente hasta que el embarazo llegue a término y comunicará inmediatamente su desenlace (ya sea normal o anómalo) al promotor.

Si el desenlace del embarazo es anómalo (por ejemplo, un aborto espontáneo o terapéutico), el investigador debe comunicarlo como AA. Si el desenlace anómalo cumple alguno de los criterios de gravedad, debe comunicarse como AAG, mediante el formulario de comunicación de AAG u otro formulario autorizado equivalente, en un plazo de 24 horas desde que el investigador tenga conocimiento del acontecimiento.

Toda muerte neonatal que tenga lugar en los 28 días posteriores al parto, independientemente de la causalidad, debe comunicarse como AAG. Asimismo, todo caso de muerte de un lactante, una vez transcurrido dicho plazo de 28 días, y si el investigador considera que guarda relación con la exposición al producto en investigación durante el embarazo, también debe comunicarse por fax u otro medio adecuado, mediante el formulario de comunicación de AAG u otro formulario autorizado equivalente, en un plazo de 24 horas desde que el investigador tenga conocimiento del acontecimiento.

9.9.1 Varones

Si la pareja mujer de un paciente varón en tratamiento con el producto en investigación se queda embarazada, el varón en tratamiento con el producto en investigación debe comunicarlo al investigador; asimismo, se indicará a su pareja embarazada que debe contactar inmediatamente con su médico.

9.9.2 Consideraciones sobre fertilidad

Basándose en datos de seguridad preclínicos, el tratamiento con cabozantinib puede afectar a la fertilidad de ambos sexos. Se debe aconsejar a los pacientes de ambos sexos que se asesoren y consideren la adopción de medidas de preservación de la fertilidad antes de iniciar el tratamiento.

10 ANÁLISIS ESTADÍSTICO

10.1 Consideraciones generales

El contenido de la base de datos será transferido a SAS© para el análisis estadístico. Todos los análisis estadísticos serán realizados usando el paquete estadístico SAS©

system para Windows versión 9.4 ó posterior. Previo al análisis estadístico, se elaborará un plan de análisis estadístico más detallado.

En caso de valores ausentes (*missings*) no se prevé realizar ninguna técnica de imputación de valores.

10.2 Análisis descriptivos

Se realizarán análisis descriptivos de las variables recogidas. Dependiendo del carácter de la variable se presentará:

- Las variables categóricas se resumirán mediante frecuencias y porcentajes.
- Las variables continuas se resumirán mediante las medidas de tendencia central y dispersión: media, desviación estándar, mediana, los percentiles del 25% y el 75% (Q1 y Q3) y valores extremos (mínimo y máximo).

10.3 Análisis bivariante

Cuando sea de interés para responder los objetivos del estudio, se evaluará la relación entre variables:

- Para dos variables categóricas se presentarán tablas de contingencia con la frecuencia en cada categoría y el porcentaje por columnas. Para evaluar la posible asociación se realizarán pruebas de Chi cuadrado o test exacto de Fisher y se presentará el p-valor resultante.
- Para una variable numérica con una categórica se presentarán los estadísticos descriptivos por grupos. Para evaluar la posible asociación se realizarán pruebas T-Test, ANOVA o las pruebas no paramétricas de Wilcoxon o Kruskal-Wallis, así su p-valor resultante.
- Para valorar la relación entre dos variables numéricas se presentará la correlación de Pearson o Spearman, así como su p-valor resultante

10.4 Evaluación de la respuesta (eficacia)

Objetivo principal

Para el análisis del objetivo principal (tasa de respuesta) se presentará el recuento y porcentaje de pacientes con cada una de las categeorias de la mejor respuesta al tratamiento (Respuesta Completa, Respuesta Parcial, Enfermedad Estable y Progresión de la Enfermedad). También se presentará el recuento y porcentaje de pacientes con respuesta completa o parcial, además se presentará el intervalo de confianza del 95% del porcentaje.

Objetivos secundarios

Para la supervivencia global y supervivencia libre de progresión se estimará una curva de supervivencia mediante el método de Kaplan-Meier y se presentarán las estimaciones de los percentiles 25, 50 (mediana) y 75, junto a sus intervalos de confianza del 95%.

Par los biomarcadores se presentará una descriptiva de los biomarcadores a lo largo del seguimiento de los pacientes, se utilizará la metodología descrita en el apartado 9.2.

10.5 Análisis intermedios

Tras la inclusión de los primeros 26 pacientes, se interrumpirá temporalmente el reclutamiento. En el momento en que los 26 pacientes hayan completado las 12 semanas de tratamiento pre-operatorio con cabozantinib se llevará a cabo un análisis intermedio, en el que se evaluará la tasa de respuesta objetiva de estos pacientes. Este análisis intermedio será realizado por la CRO contratada. Dichos resultados se revisarán con el

coordinador del estudio. En caso de que 7 o menos de los 26 pacientes alcancen una respuesta objetiva, se detendrá definitivamente el reclutamiento.

10.6 Análisis de seguridad

Se realizará un análisis descriptivo de los acontecimientos adversos registrados, nombrados según el término preferente (Preferred Term – PT), y agrupados según el órgano/sistema afectado (System Organ Class – SOC) del diccionario MedDRA (versión 17.0 o posterior).

10.7 Cálculo del tamaño de la muestra

10.7.1 Tamaño total de la muestra

Al tratarse de un ensayo clínico de fase 2 con dos estadios con un objetivo principal de valoración de la eficacia del fármaco, para el cálculo del tamaño de muestra se ha utilizado el método de *Simon's two stage* (Jung et al. 2004; Simon 1989).

Los criterios para la interrupción prematura del estudio se han establecido en base a los ensayos clínicos previos con cabozantinib en el tratamiento del cáncer de células renales. El ensayo clínico de registro METEOR reportó una tasa de respuesta objetiva cercana al 20% (Choueiri et al. 2016). En base a esta observación, se ha establecido una probabilidad de respuesta en caso de mal fármaco (p0) de 0.25. En un ensayo clínico posterior (CABOSUN) se reportó una tasa de respuesta objetiva con cabozantinib del 46% (Choueiri et al. 2017), por lo que la probabilidad de respuesta en caso de buen fármaco (p1) se ha establecido en 0.45. Asumiendo estas dos proporciones y con una potencia estadística del 90% y un error de tipo 1 (α unilateral) de 0.05, el cálculo de la muestra del estudio es el siguiente:

N	N1	R1	R2	Error de tipo 1	Potencia	EN_0	Probabilidad de interrupción prematura.
50	26	7	17	0.0498	0.90	33.6	0.69

- N: número total de sujetos.
- N1: número de sujetos reclutados durante el estadio 1.
- R1: el estudio se interrumpirá prematuramente por fracaso en caso de que se observen R1 o menos pacientes con respuesta durante el estadio 1.
- R2: en caso de que se observen R2 o menos respuestas al final del estadio 2, no estará justificada ninguna investigación adicional.
- EN_0: número de pacientes en caso de que la respuesta sea p0.

En base al cálculo del tamaño de muestra, en el primer estadio del ensayo se reclutará un total de 26 pacientes. Una vez se haya evaluado la respuesta al tratamiento en estos pacientes si el número de pacientes con respuesta parcial o completa es de 7 pacientes o inferior el ensayo se finalizará por falta de eficacia. Si el número de pacientes con respuesta parcial o completa es superior a 7, se empezará el reclutamiento del segundo estadio para el cual se reclutarán 24 pacientes más, hasta llegar a un total de 50 pacientes en el ensayo. Si al final del segundo estadio el número total de pacientes con respuesta parcial o completa es igual o inferior a 17 pacientes no estará justificada ninguna investigación adicional debido a la falta de eficacia.

10.8 Desviación del plan de análisis estadístico

Cualquier desviación de ese plan estadístico presentado será descrita y justificada en el informe final.

11 CONTROL Y GARANTÍA DE CALIDAD

11.1 Monitorización del estudio

El estudio será monitorizado por la empresa de investigación por contrato (CRO) BioClever 2005, S.L. De conformidad con las normativas aplicables y las Buenas Prácticas Clínicas (BPC), el/la monitor/a visitará o se pondrá en contacto con el centro de forma periódica. La duración, naturaleza y frecuencia de dichas visitas/contactos se establecerá en un plan de monitorización.

A lo largo de estos contactos, el/la monitor/a deberá:

- controlar y evaluar los avances del estudio;
- examinar los datos recopilados;
- llevar a cabo una verificación de los documentos fuente;
- identificar cualquier problema y encontrar soluciones;

El objetivo de la actividad de control es verificar que:

- se respetan los derechos y el bienestar de los sujetos;
- los datos del estudio son precisos, completos y verificables con la ayuda de los documentos originales;
- el estudio se lleva a cabo de acuerdo con el protocolo y con cualquier enmienda aprobada, BPC y normativas aplicables.

El investigador debe estar de acuerdo en:

- conceder al/la monitor/a acceso directo a toda la documentación relevante;
- dedicar parte de su tiempo y del tiempo de su personal al monitor con el objetivo de tratar los resultados de la supervisión, así como cualquier otro posible aspecto.

El monitor también debe ponerse en contacto con el centro antes de comenzar el estudio con el objetivo de analizar con el personal tanto el protocolo como los procedimientos de recopilación de datos.

11.2 Auditorías e inspecciones

El promotor puede llevar a cabo si lo considera oportuno una auditoría de control de calidad a su exclusiva discreción. En este caso, el investigador deberá estar de acuerdo en conceder al auditor acceso directo a toda la documentación relevante y dedicar parte de su tiempo y del tiempo de su personal al auditor con el objetivo de tratar los resultados de la supervisión, así como cualquier otro posible aspecto.

Asimismo, las autoridades sanitarias también podrán inspeccionar el estudio. En este caso, el investigador deberá estar de acuerdo en conceder al inspector acceso directo a toda la documentación relevante y dedicar parte de su tiempo y del tiempo de su personal al inspector con el objetivo de tratar los resultados de la supervisión, así como cualquier otro posible aspecto.

11.3 Cuaderno de recogida de datos

La recogida de datos se realizará mediante un CRD electrónico (CRDe) con un sistema de acceso mediante usuario y contraseña. El CRDe contendrá reglas de coherencia interna y filtros con el fin de minimizar los posibles errores de entrada de datos.

La recolección precisa y fiable de datos estará garantizada por la verificación y comprobación cruzada del CRD frente a los registros del centro, realizada por el monitor del estudio (verificación de documentos fuente). Los datos recopilados se incorporarán a una base de datos informática que será revisada para detectar posibles inconsistencias que deberá resolver el equipo investigador del estudio en cada centro.

12 ASPECTOS ÉTICOS

12.1 Consideraciones generales

Este estudio se llevará a cabo siguiendo los principios éticos básicos contenidos en la Declaración de Helsinki, (versión Fortaleza, Brasil, Octubre 2013) y según la reglamentación española (Real Decreto 1090/2015, de 4 de diciembre, por el que se regulan los ensayos clínicos con medicamentos, los Comités de Ética de la Investigación con medicamentos y el Registro Español de Estudios Clínicos). El estudio no se iniciará antes de que el Comité de Ética en la Investigación competente haya evaluado y aprobado el protocolo.

La participación de los investigadores en este estudio es libre, voluntaria e independiente. Tanto los investigadores participantes como el resto de personal involucrado en el estudio deberán cumplir las normas de BPC, tal como se describe en la Guía ICH Tripartita y Armonizada para la BPC (revisión de 2016).

Este estudio se iniciará únicamente tras las siguientes acciones:

- Aceptación del protocolo por parte del investigador y el promotor.
- Dictamen único del CEIm
- Autorización de la Agencia Española del Medicamento y Productos Sanitarios.

Se garantiza a todos los pacientes una atención médica y de enfermería durante la duración del ensayo.

12.2 Información al paciente y consentimiento informado

El investigador deberá informar al paciente de la naturaleza, duración y propósito de este estudio y, además, de todos los inconvenientes y obstáculos que, en caso de que exista alguno, pueden esperarse. Además se proporcionará información por escrito al paciente. Los pacientes participantes deben tener capacidad legal para dar su consentimiento y ejercer su libertad de decisión. El Consentimiento por escrito se obtendrá mediante el consentimiento informado antes de incluir al paciente en el estudio.

13 CONFIDENCIALIDAD Y PROTECCIÓN DE DATOS

13.1 Acceso a los datos

Los investigadores e instituciones permitirán las monitorizaciones y auditorias por parte de las autoridades sanitarias o el promotor dando acceso directo a los datos y documentos fuente originales.

El acceso a la información personal de los pacientes quedará restringido al médico del estudio/colaboradores. Para permitir monitorizaciones, auditorias e inspecciones, se garantiza el acceso a los datos a las autoridades sanitarias, al Comité Ético de Investigación con medicamentos (CEIm) y personal autorizado por el promotor, siempre manteniendo la confidencialidad de los mismos de acuerdo a la legislación vigente.

13.2 Confidencialidad

Toda la información proporcionada por el Promotor (o cualquier compañía / institución que actúe en su nombre), o producida durante el estudio, incluidos, entre otros, el protocolo, los datos personales en relación con los pacientes, el eCRF, los manuales del investigador y los resultados obtenidos durante el curso del estudio, es confidencial, antes de la publicación de los resultados.

El Investigador y cualquier persona bajo su autoridad acuerdan comprometerse a mantener la confidencialidad y no divulgar la información a ningún tercero sin la aprobación previa por escrito del Promotor.

Sin embargo, el envío de este protocolo de estudio y otra documentación necesaria al Comité de Ética está permitido, ya que sus miembros tienen la misma obligación de confidencialidad.

El personal del centro estará sujeto a la misma obligación que el investigador. El investigador deberá informar al personal del centro de la naturaleza confidencial del estudio.

13.3 Protección de Datos

Los datos recopilados en este estudio solo se usarán para los fines del estudio.

Los datos personales del paciente se tratarán de acuerdo con todas las leyes y regulaciones aplicables (Reglamento Europeo 679/2016 de protección de datos).

Al archivar o procesar datos personales pertenecientes al investigador y/o a los pacientes, el Promotor y cualquier empresa/institución que actúe en su nombre tomarán todas las medidas apropiadas para salvaguardar y evitar el acceso a estos datos por parte de terceros no autorizados.

Los datos recopilados para el estudio se identificarán mediante un código, de modo que no incluya información que pueda identificar al paciente, y solo el investigador y el personal delegado del centro podrán relacionar los datos con el paciente y su historial médico. Por lo tanto, la identidad del paciente no se revelará a ninguna persona, salvo excepciones en caso de urgencia médica o requerimiento legal.

El tratamiento, la comunicación y la asignación de los datos personales de todos los participantes se realizarán de conformidad con las disposiciones de todas las leyes y normativas aplicables.

13.4 Archivo de documentación

13.4.1 Archivos del investigador y retención de documentos

El investigador debe conservar un archivo del investigador con los registros adecuados y precisos que permitan que la realización del estudio esté plenamente documentada y que los datos del estudio sean posteriormente verificados.

El archivo de estudio del investigador contendrá el protocolo y sus enmiendas, una copia de los CRD, formularios de cuestionarios, aprobación del CEIm y autorización de las autoridades sanitarias, muestras de la hoja de información al paciente y consentimiento informado, currículum del personal, listados de identificación de firmas y listado de identificación de sujetos, así como otros documentos y correspondencia apropiada.

Los documentos clínicos fuente del sujeto (habitualmente predefinidos por el proyecto para registrar parámetros clave de eficacia y seguridad o documentos que no consten en la historia clínica del centro) se archivarán indicando el número de paciente, sin datos personales.

El investigador debe conservar estos documentos 25 años, según el RD 1090/2015, siempre que el promotor no exprese otro período de tiempo.

13.4.2 Documentos fuente y datos básicos

Se recogerá en la historia clínica la participación del paciente en el estudio, así como el número de código asignado y la identificación de las diferentes visitas que tendrán lugar a lo largo del estudio. Al finalizar el estudio, se dejará una copia en el centro de los datos almacenados en el CRD.

14 FINANCIACIÓN Y SEGUROS

14.1 Fuente de financiación

El estudio será financiado por el promotor (ONCOSUR). Los detalles de esta financiación se especificarán en la memoria económica del estudio.

14.2 Póliza de seguros

De acuerdo con lo dispuesto en el Real Decreto 1090/2015, de 4 de diciembre, el promotor del ensayo cuenta con una póliza de seguro de responsabilidad civil con la compañía HDI, con domicilio social en Madrid. El Promotor prorrogará esta póliza u otra con cobertura equivalente hasta la finalización del ensayo. La póliza cubrirá los daños y perjuicios que como consecuencia del ensayo pudieran resultar para las personas en que hubiera de realizarse. , Esta póliza cubre, asimismo, las responsabilidades del promotor, el/la investigador/a principal y sus colaboradores, así como las del Hospital o Centro donde se lleve a cabo el ensayo clínico.

El Promotor se compromete a abonar las primas correspondientes que cubran la responsabilidad civil relativa al ensayo. Se presume, salvo prueba en contrario, que los daños que afecten a la salud de la persona sujeta a ensayo durante su realización y en el año siguiente a la terminación del tratamiento, se han producido como consecuencia del ensayo. Sin embargo, una vez concluido el año, el sujeto del ensayo está obligado a probar el nexo entre el ensayo y el daño producido.

El Centro y el investigador principal se comprometen a informar al Promotor de cualquier reclamación o acción legal, real o potencial si es conocida, vinculable al ensayo.

15 POLÍTICA DE PUBLICACIÓN

Está previsto divulgar los resultados que sean de interés médico en congresos nacionales e internacionales, así como en revistas "peer-review". Los artículos publicados en revistas se redactarán de acuerdo a las buenas prácticas de publicación GPP3 (*Principles of Good Publication Practice for Company-Sponsored Medical Research*) y siguiendo las recomendaciones del comité internacional de editores de revistas médicas (ICMJE).

Cualquier presentación formal o publicación de los datos de este estudio será considerada como una publicación conjunta por el(los) investigador(es) junto al Promotor. Los coordinadores científicos del estudio ocuparán una posición preferente en el orden de autoría de las publicaciones derivadas del estudio.

16 REFERENCIAS

AEMPS. 2010. “Anexo 13 - Fabricación de Medicamentos En Investigación.” *Guía de Normas de Correcta Fabricación de Medicamentos de Uso Humano Y Veterinario*. https://www.aemps.gob.es/industria/inspeccionNCF/guiaNCF/docs/anexos/26_anexo-13.pdf.

Bellmunt, J, J Puente, J G de Muro, N Lainez, C Rodriguez, and I Duran. 2014. “SEOM Clinical Guidelines for the Treatment of Renal Cell Carcinoma.” *Clinical & Translational Oncology* 16 (12). Springer: 1043–50. doi:10.1007/s12094-014-1219-1.

Bellmunt, Joaquim, Emiliano Calvo, Daniel Castellano, Miguel Ángel Climent, Emilio Esteban, Xavier García del Muro, José Luis González-Larriba, Pablo Maroto, and José Manuel Trigo. 2014. “Recommendations from the Spanish Oncology Genitourinary Group for the Treatment of Patients with Renal Cell Carcinoma.” *Cancer Chemother Pharmacol* 73 (6): 1095–1107. doi:10.1007/s00280-009-0955-3.

Chapin, Brian F., Scott E. Delacroix, Stephen H. Culp, Graciela M Nogueras Gonzalez, Nizar M. Tannir, Eric Jonasch, Pheroz Tamboli, and Christopher G. Wood. 2011. “Safety of Presurgical Targeted Therapy in the Setting of Metastatic Renal Cell Carcinoma.” *European Urology* 60 (5): 964–71. doi:10.1016/j.eururo.2011.05.032.

Chavan, Saurabh, Freddie Bray, Joannie Lortet-Tieulent, Michael Goodman, and Ahmedin Jemal. 2014. “International Variations in Bladder Cancer Incidence and Mortality.” *European Urology* 66 (1). Elsevier: 59–73.

Choueiri, Toni K., André P. Fay, Robert Gagnon, Ying Lin, Brittany Bahamon, Victoria Brown, Jonathan E. Rosenberg, et al. 2013. “The Role of Aberrant VHL/HIF Pathway Elements in Predicting Clinical Outcome to Pazopanib Therapy in Patients with Metastatic Clear-Cell Renal Cell Carcinoma.” *Clinical Cancer Research* 19 (18): 5218–26. doi:10.1158/1078-0432.CCR-13-0491.

Choueiri, Toni K, Bernard Escudier, Thomas Powles, Paul N Mainwaring, Brian I Rini, Frede Donskov, Hans Hammers, Thomas E Hutson, Jae-Lyun Lee, and Katriina Peltola. 2015. “Cabozantinib versus Everolimus in Advanced Renal-Cell Carcinoma.” *New England Journal of Medicine* 373 (19). Mass Medical Soc: 1814–23.

Choueiri, Toni K, Bernard Escudier, Thomas Powles, Nizar M Tannir, Paul N Mainwaring, Brian I Rini, Hans J Hammers, et al. 2016. “Cabozantinib versus Everolimus in Advanced Renal Cell Carcinoma (METEOR): Final Results from a Randomised, Open-Label, Phase 3 Trial.” *The Lancet Oncology* 17 (7): 917–27. doi:10.1016/S1470-2045(16)30107-3.

Choueiri, Toni K, Susan Halabi, Ben L Sanford, Olwen Hahn, M Dror Michaelson, Meghara K Walsh, Darren R Feldman, et al. 2017. “Cabozantinib versus Sunitinib as Initial Targeted Therapy for Patients with Metastatic Renal Cell Carcinoma of Poor or Intermediate Risk: The Alliance A031203 CABOSUN Trial.” *Journal of Clinical Oncology* 35 (6). American Society of Clinical Oncology: 591–97. doi:10.1200/JCO.2016.70.7398.

Cockcroft, Donald W, and Henry Gault. 1976. “Prediction of Creatinine Clearance from Serum Creatinine.” *Nephron* 16 (1). Karger Publishers: 31–41.

CTFG. 2014. “Clinical Trial Facilitation Group: Recommendations Related to Contraception and Pregnancy Testing in Clinical Trials.” http://www.hma.eu/fileadmin/dateien/Human_Medicines/01-About_HMA/Working_Groups/CTFG/2014_09_HMA_CTFG_Contraception.pdf.

Eble, John N. 2004. *World Health Organization Classification of Tumours*. *Pathology and Genetics. Tumours of the Urinary System and Male Genital Organs*. Vol. 10. IARC press.

Eisenhauer, E. A., P. Therasse, J. Bogaerts, L. H. Schwartz, D. Sargent, R. Ford, J. Dancey, et al. 2009. “New Response Evaluation Criteria in Solid Tumours: Revised

RECIST Guideline (Version 1.1).” *European Journal of Cancer* 45 (2). Elsevier: 228–47. doi:10.1016/j.ejca.2008.10.026.

Flanigan, Robert C, Sydney E Salmon, Brent A Blumenstein, Scott I Bearman, Vivek Roy, Patrick C McGrath, John R Caton Jr, Nikhil Munshi, and E David Crawford. 2001. “Nephrectomy Followed by Interferon Alfa-2b Compared with Interferon Alfa-2b Alone for Metastatic Renal-Cell Cancer.” *New England Journal of Medicine* 345 (23). Mass Medical Soc: 1655–59.

Hanna, Nawar, Maxine Sun, Christian P. Meyer, Paul L. Nguyen, Sumanta K. Pal, Steven L. Chang, Guillermo De Velasco, Quoc Dien Trinh, and Toni K. Choueiri. 2016. “Survival Analyses of Patients with Metastatic Renal Cancer Treated with Targeted Therapy with or without Cytoreductive Nephrectomy: A National Cancer Data Base Study.” *Journal of Clinical Oncology* 34 (27): 3267–75. doi:10.1200/JCO.2016.66.7931.

Heng, Daniel Y.C., J. Connor Wells, Brian I. Rini, Benoit Beuselinck, Jae Lyun Lee, Jennifer J. Knox, Georg A. Bjarnason, et al. 2014. “Cytoreductive Nephrectomy in Patients with Synchronous Metastases from Renal Cell Carcinoma: Results from the International Metastatic Renal Cell Carcinoma Database Consortium.” *European Urology* 66 (4): 704–10. doi:10.1016/j.eururo.2014.05.034.

Jung, Sin Ho, Taiyeong Lee, Kyung Mann Kim, and Stephen L. George. 2004. “Admissible Two-Stage Designs for Phase II Cancer Clinical Trials.” *Statistics in Medicine* 23 (4): 561–69. doi:10.1002/sim.1600.

Kim, Jung Han, Bum Jun Kim, and Hyeong Su Kim. 2017. “Clinicopathological Impacts of High c-Met Expression in Renal Cell Carcinoma : A Meta-Analysis and Review.” *Oncotarget* 8 (43): 75478–87. doi:10.18632/oncotarget.20796.

Ko, Jenny J, Wanling Xie, Nils Kroeger, Jae lyun Lee, Brian I Rini, Jennifer J Knox, Georg A Bjarnason, et al. 2015. “The International Metastatic Renal Cell Carcinoma Database Consortium Model as a Prognostic Tool in Patients with Metastatic Renal Cell Carcinoma Previously Treated with First-Line Targeted Therapy: A Population-Based Study.” *The Lancet Oncology* 16 (3). Elsevier: 293–300. doi:10.1016/S1470-2045(14)71222-7.

Koneru, R, and S J Hotte. 2009. “Role of Cytokine Therapy for Renal Cell Carcinoma in the Era of Targeted Agents.” *Current Oncology (Toronto, Ont.)* 16 (Suppl 1). Multimed Inc.: S40–44. doi:10.3747/co.v16i0.417.

Mickisch, G H J, A Garin, Hendrik Van Poppel, Linda de Prijck, and Richard Sylvester. 2001. “Radical Nephrectomy plus Interferon-Alfa-Based Immunotherapy Compared with Interferon Alfa Alone in Metastatic Renal-Cell Carcinoma: A Randomised Trial.” *The Lancet* 358 (9286). Elsevier: 966–70.

Motzer, Robert J, Jennifer Bacik, Barbara A Murphy, Paul Russo, and Madhu Mazumdar. 2002. “Interferon-Alfa as a Comparative Treatment for Clinical Trials of New Therapies against Advanced Renal Cell Carcinoma.” *Journal of Clinical Oncology : Official Journal of the American Society of Clinical Oncology* 20 (1). American Society of Clinical Oncology: 289–96. doi:10.1200/jco.2002.20.1.289.

Motzer, Robert J, Bernard Escudier, David F McDermott, Saby George, Hans J Hammers, Sandhya Srinivas, Scott S Tykodi, Jeffrey A Sosman, Giuseppe Procopio, and Elizabeth R Plimack. 2015. “Nivolumab versus Everolimus in Advanced Renal-Cell Carcinoma.” *New England Journal of Medicine* 373 (19). Mass Medical Soc: 1803–13.

Motzer, Robert J, Eric Jonasch, Neeraj Agarwal, Clair Beard, Sam Bhayani, Graeme B Bolger, Sam S Chang, Toni K Choueiri, Brian A Costello, and Ithaar H Derweesh. 2015. “Kidney Cancer, Version 3.2015.” *Journal of the National Comprehensive Cancer Network* 13 (2). Harborside Press, LLC: 151–59.

National Cancer Institute. 2018. “Cancer Therapy Evaluation Program.” https://ctep.cancer.gov/protocoldevelopment/electronic_applications/ctc.htm#ctc_40.

Posadas, Edwin M., Suwicha Limvorasak, Shaleekha Sharma, and Robert A. Figlin. 2013.

"Targeting Angiogenesis in Renal Cell Carcinoma." *Expert Opinion on Pharmacotherapy* 14 (16): 2221–36. doi:10.1517/14656566.2013.832202.

Powles, Thomas, Michael Staehler, Börje Ljungberg, Karim Bensalah, Steven E Canfield, Saeed Dabestani, Rachel Giles, Fabian Hofmann, Milan Hora, and Markus A Kuczyk. 2016. "Updated EAU Guidelines for Clear Cell Renal Cancer Patients Who Fail VEGF Targeted Therapy." *European Urology* 69 (1). Elsevier: 4–6.

Puiggròs, C, M Lecha, T Rodríguez, C Pérez-Portabella, and M Planas. 2009. "Karnosfsky Index as a Mortality Predicting Factor in Patients on Home-Based Enteral Nutrition." *Nutricion Hospitalaria* 24 (2): 156–60.

Sierra, J Rafael, and Ming Sound Tsao. 2011. "C-MET as a Potential Therapeutic Target and Biomarker in Cancer." *Therapeutic Advances in Medical Oncology*. SAGE Publications. doi:10.1177/1758834011422557.

Simon, Richard. 1989. "Optimal Two-Stage Designs for Phase II Clinical Trials." *Controlled Clinical Trials* 10 (1): 1–10. doi:10.1016/0197-2456(89)90015-9.

Sobin, L H, and M K Gospodarowicz. 2009. "Wittekind Ch. Eds. TNM Classification of Malignant Tumors." In *UICC International Union Against Cancer*, 7th Editio, 255–57. wiley-blackwell, Oxford.

Stroup, Sean P., Omer A. Raheem, Kerrin L. Palazzi, Michael A. Liss, Reza Mehrazin, Ryan P. Kopp, Nishant Patel, et al. 2013. "Does Timing of Cytoreductive Nephrectomy Impact Patient Survival with Metastatic Renal Cell Carcinoma in the Tyrosine Kinase Inhibitor Era? A Multi-Institutional Study." *Urology* 81 (4): 805–11. doi:10.1016/j.urology.2012.10.054.

Sun, Maxine, Guillermo De Velasco, Quoc Dien Trinh, and Toni K. Choueiri. 2016. "Assessing the Contemporary Role of Cytoreductive Nephrectomy in Metastatic Renal Cell Carcinoma: Another Step in the Right Direction." *European Urology*, June. doi:10.1016/j.eururo.2015.05.040.

Yakes, F Michael, Jason Chen, Jenny Tan, Kyoko Yamaguchi, Yongchang Shi, Peiwen Yu, Fawn Qian, Felix Chu, Frauke Bentzien, and Belinda Cancilla. 2011. "Cabozantinib (XL184), a Novel MET and VEGFR2 Inhibitor, Simultaneously Suppresses Metastasis, Angiogenesis, and Tumor Growth." *Molecular Cancer Therapeutics* 10 (12). AACR: 2298–2308.

Znaor, Ariana, Joannie Lortet-Tieulent, Mathieu Laversanne, Ahmedin Jemal, and Freddie Bray. 2015. "International Variations and Trends in Renal Cell Carcinoma Incidence and Mortality." *European Urology* 67 (3). Elsevier: 519–30.

ANEXO I: DEFINICIONES PARA EL ESTADIAJE DEL CÁNCER DE CÉLULAS RENALES DE ACUERDO CON LA CLASIFICACIÓN TNM DEL UICC

La unión internacional contra el cáncer, en su consenso de 2009, establece las siguientes definiciones para el tamaño, invasión de ganglios linfáticos y metástasis:

Tamaño

- T1: Tumor ≤ 7.0 cm de diámetro y limitado al riñón.
- T1a: Tumor ≤ 4.0 cm de diámetro y limitado al riñón.
- T1b: Tumor de > 4.0 cm (pero ≤ 7.0 cm) de diámetro y limitado al riñón.
- T2: tumor > 7.0 cm de diámetro y limitado al riñón.
- T2a: Tumor de > 7.0 cm (pero ≤ 10 cm) de diámetro y limitado al riñón.
- T2b: Tumor de > 10 cm de diámetro.
- T3: El tumor se extiende a los vasos principales o invade la glándula suprarrenal o los tejidos circundantes, sin invadir la fascia de Gerota.
- T3a: El tumor invade los tejidos periféricos, sin invadir la fascia de la Gerota.
- T3b: El tumor se extiende a la vena cava por debajo del diafragma.
- T3c: El tumor se extiende a la vena cava por encima del diafragma o invade la pared de la vena cava.
- T4: El tumor invade más allá de la fascia de Gerota.

Ganglios linfáticos locales

- N0: Ausencia de metástasis en ganglios linfáticos.
- N1: Metástasis en uno o más ganglios linfáticos.

Metástasis a distancia

- M0: no metástasis.
- M1: metástasis a distancia.

Fuente:

Sobin LH, Gospodariwicz M, Wittekind C (eds). TNM classification of malignant tumors. UICC International Union Against Cancer. 7th edn. Wiley-Blackwell, 2009: pp. 255-257.

ANEXO II: ESCALAS DE VALORACIÓN FUNCIONAL

Escala de valoración funcional del Eastern Cooperative Oncology Group (ECOG):

Grado	
0	Totalmente activo, capaz de llevar a cabo sin limitaciones todas las actividades anteriores a la enfermedad.
1	Restricción de las actividades físicamente agotadoras, pero la persona puede caminar y efectuar tareas de naturaleza sedentaria o reposada, p. ej. tareas domésticas o de oficina ligeras.
2	Capaz de caminar y ocuparse de su cuidado personal, pero no puede llevar a cabo ninguna actividad laboral. Puede deambular más del 50 % del tiempo.
3	Solo es capaz de cuidar de sí mismo de forma limitada, está confinado a una cama o una silla durante más del 50 % del periodo de vigilia.
4	Completamente incapacitado. No puede ocuparse de ningún tipo de cuidado personal. Totalmente confinado a una cama o una silla.
5	Muerte

Índice de Karnofsky

Puntuación	Situación clínico-funcional
100	Normal, sin quejas ni evidencia de enfermedad.
90	Capaz de llevar a cabo actividad normal pero con signos o símptomas leves.
80	Actividad normal con esfuerzo, algunos signos y síntomas de enfermedad.
70	Capaz de cuidarse, pero incapaz de llevar a cabo actividad normal o trabajo activo.
60	Requiere atención ocasional, pero es capaz de satisfacer la mayoría de sus necesidades.
50	Necesita ayuda importante y asistencia médica frecuente.
40	Incapaz, necesita ayuda y asistencia especiales.
30	Totalmente incapaz, necesita hospitalización y tratamiento de soporte activo.
20	Muy gravemente enfermo, necesita tratamiento activo.
10	Moribundo irreversible.
0	Muerto

ANEXO III: CLASIFICACIÓN CLAVIEN-DINDO DE COMPLICACIONES QUIRÚRGICAS

Grados	Definiciones
I	Cualquier desviación del curso postoperatorio normal sin la necesidad de tratamiento farmacológico o intervenciones quirúrgicas, endoscópicas y radiológicas<br>Los regímenes terapéuticos aceptables son los medicamentos como los antieméticos, antipiréticos, analgésicos, diuréticos y electrolitos y la fisioterapia. Este grado también incluye las infecciones de la herida abierta en la cabecera del paciente
II	Requiere tratamiento farmacológico con medicamentos distintos de los autorizados para las complicaciones de grado I. También se incluyen las transfusiones de sangre y la nutrición parenteral total
III	Requiere intervención quirúrgica, endoscópica o radiológica
IIIa	Intervención que no se da bajo anestesia general
IIIb	Intervención bajo anestesia general
IV	Complicación potencialmente mortal (incluidas las complicaciones del sistema nervioso central: hemorragia cerebral, infarto cerebral, hemorragia subaracnoidea, pero con exclusión de los ataques isquémicos transitorios) que requiere de la gestión de la Unidad de Cuidados Intermedios/Intensivos
IVa	Disfunción de un solo órgano (incluyendo la diálisis)
IVb	Disfunción multiorgánica
V	Muerte de un paciente
Sufijo «d»	Si el paciente padece una complicación en el momento del alta se añade el sujijo «d» (de discapacidad) al respectivo grado de complicación. Esta etiqueta indica la necesidad de seguimiento para evaluar la complicación al completo

Fuente de la versión Española:

Mitropoulos D, Artibani W, Graefen M, Remzi M, Rouprêt M, Truss M. Notificación y clasificación de complicaciones después de procedimientos quirúrgicos urológicos: una evaluación y recomendaciones del panel de guías clínicas de la EAU. Actas Urológicas Españolas. 2013 Jan 1;37(1):1-1.

ANEXO IV: LISTADO DE CENTROS E INVESTIGADORES

Dr. Guillermo de Velasco
Hospital Universitario 12 de Octubre
Av. Cordoba, s/n, 28041 Madrid

Dr. Juan F. Rodríguez
Centro Integral Oncológico Clara Campal del Hospital HM Universitario Sanchinarro.
Calle de Oña, 10, 28050 Madrid

Dr. Álvaro Pinto
Hospital Universitario La Paz
Paseo de la Castellana, 261, 28046 Madrid

Dra. Teresa Alonso
Hospital Universitario Ramón y Cajal
Ctra. De Colmenar Viejo, km. 9,100, 28034 Madrid

Dra. Ana López Martín
Hospital Universitario Severo Ochoa. Leganes
Av. de Orellana, s/n, 28911 Leganés, Madrid

Dr. Xavier García del Muro
Institut Català d'Oncologia, IDIBELL
Av. Gran Via de L'Hospitalet 199-203, 08908 L'Hospitalet de Llobregat

Dr. Emilio Esteban
Hospital Universitario Central de Asturias
Av. Roma, s/n, 33011 Oviedo

Dr. Ignacio Durán
Hospital Universitario de Valdecilla
Av. Valdecilla, 25, 39008 Santander
